# Consensus-based recommendations for the rehabilitation of children with arthrogryposis multiplex congenita: an integrated knowledge translation approach

**DOI:** 10.1186/s13023-025-03671-x

**Published:** 2025-04-09

**Authors:** Noémi Dahan‑Oliel, Sarah Cachecho, Clarice Araujo, Alicja Fąfara, Francis Lacombe, Ani Samargian, Camille Costa, Maureen Donohoe, Ann Flanagan, Bart Kowalczyk, Courtney Krakie, Lisa Wagner, Carolina Navalón, Verity Pacey, Unni Steen, Misha Walker, Trudy Wong, André Bussières

**Affiliations:** 1 Shriners Hospital for Children-Canada, 1003, Boulevard Décarie, Montréal, QC H4A 0A9 Canada; 2 School of Physical and Occupational Therapy, Faculty of Medicine and Health Sciences, McGill University, Montréal, QC Canada; 3 Institute of Physiotherapy, Jagiellonian University Medical College, Kraków, Poland; 4 Centre de Réadaptation Lucie-Bruneau, Montréal, Canada; 5 AMCSupport Inc, Spartanburg, SC USA; 6 School of Rehabilitation, Université de Montréal, Montréal, QC Canada; 7 Nemours/Alfred I duPont Hospital for Children, Delaware, USA; 8 Shriners Children’s- Chicago, Chicago, USA; 9 Department of Orthopedics, University Children’s Hospital, Kraków, Poland; 10 Shriners Children’s-Northern California, Sacramento, USA; 11 Shriners Children’s-Greenville, Greenville, USA; 12 Asociación Artrogriposis Múltiple Congénita España, Madrid, Spain; 13 Faculty of Medicine, Health and Human Sciences, Macquarie University, Sydney, Australia; 14 TRS National Resource Centre for Rare Disorders, Sunnaas Rehabilitation Hospital, Alværn, Norway; 15 The Dream Walker Project, Lima, Peru; 16 Département de Chiropratique, Université du Québec à Trois-Rivières, Trois-Rivières, QC Canada

**Keywords:** Rare diseases, Arthrogryposis multiplex congenita, Rehabilitation, Consensus-based recommendations, Integrated knowledge translation, Modified Delphi, Pediatrics

## Abstract

**Background:**

Arthrogryposis multiplex congenita (AMC) is a group of rare disorders characterized by multiple joint contractures present at birth. Early rehabilitation is essential to minimize joint contractures and maximize autonomy and participation among individuals with AMC. However, there is little robust scientific evidence to inform best practice. This project aimed to develop consensus-based recommendations for the rehabilitation management of children with AMC in the following priority areas: early intervention and motor development, muscle and joint function, orthotics, mobility, participation in areas of life, pain, psychosocial wellbeing, and perioperative rehabilitation.

**Results:**

This multi-phase project used an integrated knowledge translation approach. Based on the results from scoping reviews on the priority areas identified for the rehabilitation of children with AMC, and a clinician survey describing current practices in AMC rehabilitation, three panels of expert clinicians in occupational therapy, physical therapy, orthopedics, physiatry, and social work, as well as people with lived experience and researchers from 10 countries developed consensus-based recommendations for rehabilitation, in concordance with the Grading of Recommendations, Assessment, Development and Evaluations framework (GRADE) criteria. A modified Delphi process was completed with a wider group of international AMC experts to revise and validate the recommendations (Round 1 = 41 and Round 2 = 37 experts). A five-member external review panel appraised the recommendations using the Appraisal of Guidelines for Research and Evaluation II (AGREE II) tool. The final 16 recommendations reached a mean agreement rate of 96.6% after two Delphi rounds. The overall quality was rated at 96.6% on the AGREE II tool. Interviews with clinicians and managers identified facilitators and barriers to implementation of the recommendations in practice using the Theoretical Domain Framework.

**Conclusion:**

Consensus-based, expert validated recommendations for the rehabilitation of children with AMC were developed by a wide range of stakeholders, healthcare users and providers. The proposed recommendations are expected to contribute to improving child- and family-centered practice and health outcomes. Future work includes a knowledge translation strategy to promote sharing and implementation of the recommendations in practice.

**Supplementary Information:**

The online version contains supplementary material available at 10.1186/s13023-025-03671-x.

## Background

Arthrogryposis Multiplex Congenita (AMC) is a group of conditions that present with joint contractures in two or more body areas, affecting 1/3000 to 1/56,000 live births depending on the region surveyed, classification and coding used [1]. Causes are variable and may include genetic, parental, and environmental factors, as well as abnormalities during fetal development [2, 3]. Individuals with AMC may have limitations in range of motion in different body areas, affecting mobility, participation in daily activities and leisure, as well as effects on psychosocial wellbeing [4, 5]. Depending on the underlying diagnosis, other body systems such as the central nervous system and respiratory system may be affected [6]. Children with AMC frequently need several orthopedic surgeries to correct deformities and early intensive rehabilitation is crucial to minimize the extent of joint contractures and maximize function [7].

Throughout their career, rehabilitation practitioners may encounter only a few individuals with AMC who may significantly differ in their clinical presentation and needs. Our preliminary work with rehabilitation practitioners and youth with AMC has identified a need for the development of rehabilitation guidelines for the care of children with AMC and identified priority areas for rehabilitation [8]. Stakeholders (i.e., youth with AMC, parents, and clinicians) further validated these rehabilitation needs and priorities at the July 2017 annual AMC support group (AMCSI) meeting in Las Vegas by ranking and rating their importance. Those priorities included muscle and joint function, pain, mobility, self-care, participation and psychosocial wellbeing. Our team also undertook a knowledge synthesis consisting of a series of scoping reviews on the identified priority areas for rehabilitation [9–12]. The results from these scoping reviews revealed a lack of high-quality studies to support clinicians’ decision when choosing suitable measures and best rehabilitation care. These findings are supported by our French colleagues’ extensive 2021 review of the literature on the diagnosis and management of AMC [13].

When high-quality evidence is lacking to guide clinical decisions, clinician expertise and lived experience are important to inform best practice [14]. The role of people with lived experience in research, including those with AMC and/or family representatives, is important to facilitate the research process, help in sharing and applying the results, create partnerships, and ensures client-centeredness [15, 16], making them a valuable research partner.

The objective of this project was to develop consensus-based recommendations using a comprehensive review of the scientific literature and a consensus approach to inform rehabilitation practitioners on the management of children with AMC [17]. These consensus-based recommendations target rehabilitation practitioners—physical therapists, occupational therapists, kinesiologists, social workers, clinical specialists, public and private health practitioners, in all care settings that aim to provide rehabilitation for children with AMC, as well as program managers, policymakers (i.e., individuals at a level of government or decision-making institutions), and children with AMC, youth and their families.

## Methods

The consensus-based recommendations on the rehabilitation of children with AMC were developed using an integrated knowledge translation (iKT) multi-phase approach, involving clinicians from rehabilitation, social work, physiatry, orthopedic surgery, and individuals with lived experience. iKT is a collaborative model of research that includes knowledge users, such as care providers, patient and family representatives, and decision and policy makers, as research partners helping provide a better understanding of the problem, the environment and context where the research will be used, as well as potential barriers to dissemination and implementation, ensuring the outcomes are in line with priorities of end users [18]_._ We obtained site approval from the Shriners Hospitals for Children (CAN2004) and ethics approval from the institutional review board of the Faculty of Medicine of McGill University (A03- E51-20B).

In 2019, an advisory group composed of experts in rehabilitation, research, and lived experience (ND-O, SC, AF, FL, AS, AB) was created to inform the methodology for the development of rehabilitation recommendations. The advisory group identified its objectives, synthesized the evidence (Supplementary file 1) and facilitated the development of recommendations. The key question guiding this work was: “What are the best practices on the rehabilitation management of children with AMC in the following priority areas: early intervention and motor development, muscle and joint function, orthotics, mobility, participation in areas of life, pain, psychosocial wellbeing, and perioperative rehabilitation?”.

**Fig. 1:**
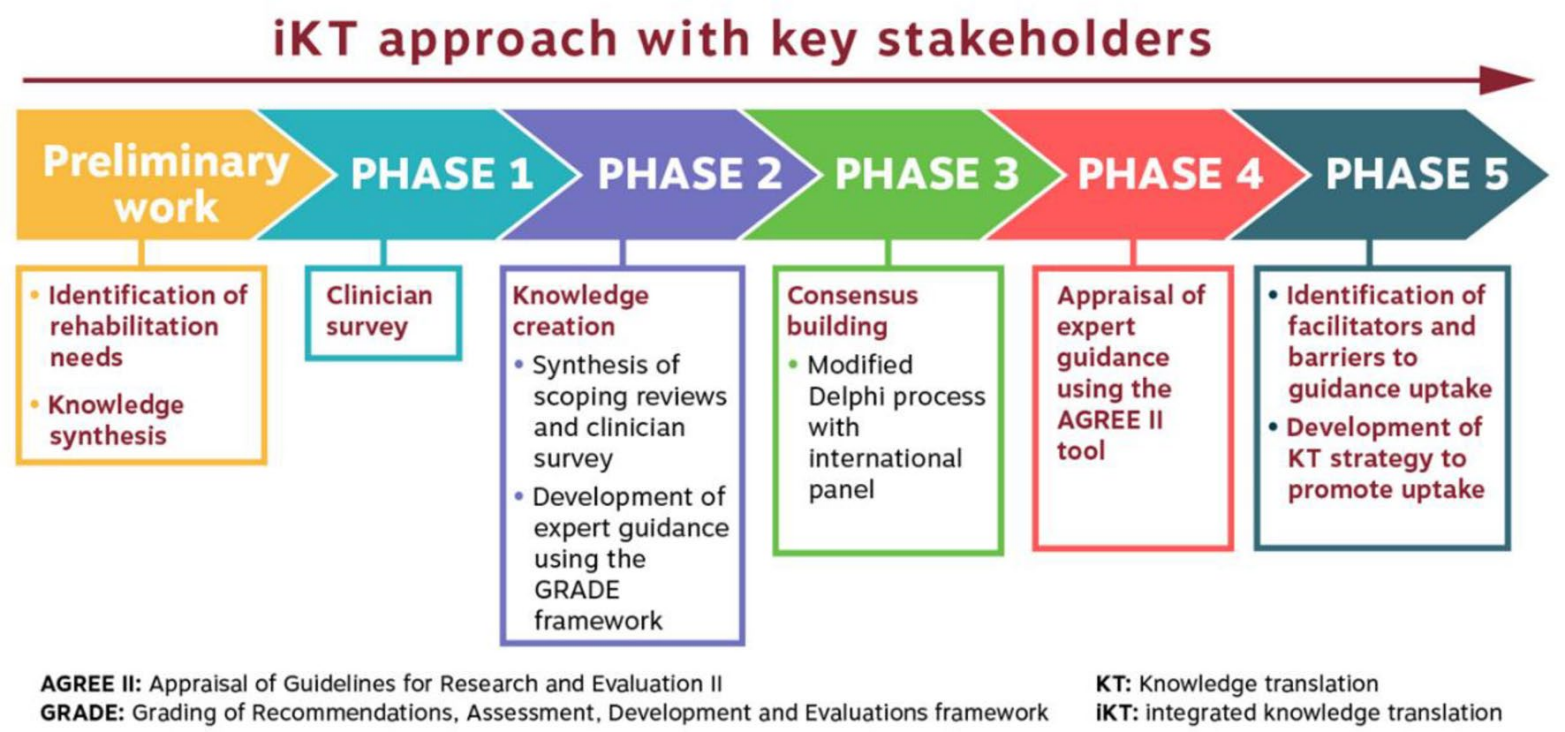
Integrated knowledge translation (iKT) multi-phase project for the development of rehabilitation recommendations for children with AMC [17]. Legend: IKT approach involving individuals with lived experience, health care practitioners and researchers, in all phases of the research process

To address this key question, we used a five-phase approach: Phase 1. Clinician survey on current practice; Phase 2. Developing recommendations; Phase 3. Consensus building; Phase 4. External appraisal; Phase 5. Facilitators and barriers to uptake of recommendations (Fig. 1). This methodology was developed in concordance with the Grading of Recommendations, Assessment, Development and Evaluations framework (GRADE) criteria [17, 19]. GRADE is a systematic, transparent and explicit approach used to develop statements that can help with decision making regarding a treatment for a specific population. GRADE served to guide the process for the development of the recommendations based on research evidence, values and preferences of end users, to facilitate implementation, adaptation to different contexts and updating of statements over time [19].

### Phase 1. Clinician survey: describing current clinical practice

The advisory group conducted an electronic survey to describe the current clinical practice of rehabilitation practitioners across different countries on the evaluation and treatment of children with AMC. The details of the methodology used in this study are described elsewhere [20]. The findings from this survey along with the scoping reviews informed the next phase.

### Phase 2. Developing recommendations

The advisory group established three panels to address different topic areas selected from the priorities previously established: “lower limbs and mobility”, “upper limbs and self-care”, and “pain, participation and psychosocial wellbeing”. Researchers, experts in rehabilitation, orthopedics, physiatry and social work with at least 5 years of experience working with children with AMC identified from Phase 1, as well as people with lived experience were invited to participate in the panels (Table 1). All panel meetings were led by a guideline development expert (AB) together with the project leads (SC, ND-O) who coordinated the meetings and prepared the content for discussions. Each panel met weekly for a 90-min meeting on Microsoft Teams®, over a 5–6 weeks period in 2021.

**Table 1:** Panel group meetings held in 2021

Topic area	Stakeholders (n)	Countries	Meetings (n)	Dates in 2021
Lower limbs and mobility	3 PTs	USA	6	February 15, 22 March 1, 8, 15, 22
	1 OT	Poland		
	1 Orthopedic surgeon	Australia		
	2 People with lived experience	Canada		
	2 Researchers			
Upper limbs and self-care	3 OTs	Canada	5	May 13, 20, 27 June 10, 17
	1 PT	USA		
	1 Orthopedic surgeon	Poland		
	2 People with lived experience	Norway		
	2 Researchers	Spain		
Pain, participation and psychosocial well-being	2 PTs	Canada	5	October 4, 12, 19, 24 November 2
	1 OT	USA		
	2 Social Workers	Poland		
	2 People with lived experience	Peru		
	1 Physiatrist			
	2 Researchers			

*PT* physiotherapist, *OT* occupational therapist, *USA* United States of America

Team members received preparatory documents one week before the first meeting, including a summary of the literature from the scoping reviews [9–12] and a summary of the results of the clinician survey [20]. The summary of the literature included levels of evidence of the included studies in the scoping reviews [9–12]. Levels of evidence were determined using the Levels of Evidence for Primary Research guidelines by the Center for Evidence-based medicine [21]. The research evidence consisted of observational studies (i.e., cohort, case series, case studies) and was considered low quality (Supplementary file 1). This excluded the possibility of quantifying the effect of the different interventions. As such, the GRADE approach was adapted to consider the experience and expertise of the panel members, in addition to the research evidence. When little research evidence pertaining to a specific topic was available, review articles were considered and indirect evidence was sought to support discussions and inform recommendations.

The panels first discussed whether the problem at hand was urgent and important. Then, they identified pertinent outcomes, related importance and level of priority. The panels then discussed interventions targeting the identified outcomes, weighting the balance of desirable (e.g., improved muscle and joint function, mobility, autonomy) and undesirable effects (e.g., fatigue, pain), as well as aspects of suitability, feasibility, clinical relevance and costs of treatment interventions.

The panel members drafted the recommendations using an iterative approach, extensively discussing them until panel members reached consensus on content, and wording, leading to the 16 recommendations. For each recommendation, details and information supplementing the statements were summarized in a paragraph accompanying each recommendation statement.

### Phase 3. Consensus building

To ensure the generalizability and acceptance of recommendations, and to improve the likelihood of uptake in clinical practice, we sought input from people with lived experience and from a wide range of expert clinicians from different countries, across different health disciplines and working in different settings. A modified Delphi process was used as this method overcomes geographical barriers and allows participants to remain anonymous and have equal opportunity to share without bias [22]. Those who completed the clinician survey (Phase 1) and expressed an interest in participating in subsequent phases of the project were invited. Other potential participants were identified through contacts previously established by the research team. The modified Delphi process consisted of an online survey sent through email with a link to the Qualtrics platform, where the survey was housed. The survey presented the different recommendations, with a summary of the additional information supplementing each recommendation or group of recommendations.

For each statement, participants were asked to rate their level of agreement on a slider scale from 0 (strongly disagree) to 100 (strongly agree). A free text space was provided for comments regarding the statement content, wording, and the additional information provided. The two rounds of survey took place between April and October 2022. A statement was kept if it met ≥ 80% agreement and could be slightly modified based on participants’ comments. For the second round of survey, those who participated in round 1 were invited and a summary of round 1 results (average agreement and standard deviation for each recommendation) was provided. In all rounds, demographic data of participants (e.g., profession, country, and years of experience and, workplace) were collected.

### Phase 4. External appraisal

In order to ensure a complete and rigorous appraisal process, the Appraisal of Guidelines for Research & Evaluation II (AGREE-II) instrument was used to appraise the rehabilitation recommendations [23]. First, the Reporting Items for practice Guidelines in HealThcare—RIGHT checklist [24, 25] was used to organize the presentation of the recommendations document and to report on its quality. Then, five independent experts not involved in the previous phases were invited to appraise the rehabilitation recommendations using the AGREE-II instrument. Individuals with expertise in pediatric rehabilitation, knowledge translation, and/or lived experience were identified from contacts known to the advisory group. The reviews took place between June and August 2023. Reviewers received the AGREE II manual, the complete recommendations document and accompanying materials (scoping reviews) and were asked to complete the online training tools recommended by the AGREE collaboration/consortium before conducting appraisals. They received a link to access the Qualtrics Platform which housed all 23 items of the AGREE-II instrument divided into its 6 domains: score and purpose; stakeholder involvement; rigour of development; clarity of presentation; applicability; editorial independence. The items were scored on a 1–7 scale (1 = strongly disagree to 7 = strongly agree). AGREE-II percentage scores for each domain were calculated with a formula provided in the manual. Based on the literature and the AGREE-II manual, high quality guidelines are those with domain scores that have a percentage score higher than 70%. Items scoring below three (i.e., disagree) were reviewed; suggestions were taken into consideration and changes were made when applicable.

### Phase 5. Facilitators and barriers to uptake of recommendations

To identify the facilitators and barriers among clinicians and managers about the uptake of the recommendations for rehabilitation of children with AMC in practice, individual interviews were conducted with a convenience sample of 15 clinicians working with children with AMC and four pediatric clinical managers. The Theoretical Domains Framework (TDF) was used to frame the interview guide for data collection, as well as to guide the analysis process. Interviews were transcribed verbatim and analyzed by four independent reviewers using a deductive thematic analysis using the TDF, followed by inductive coding. The details of the methodology used and the findings of this study are described elsewhere [26]. Identifying facilitators and barriers to knowledge uptake will help to inform a KT strategy to promote the implementation of the consensus-based recommendations for rehabilitation in practice.

## Results

This section focuses on the development of the rehabilitation recommendations using the panels of experts, consensus-building with two Delphi rounds and the external appraisal using the AGREE-II, which are detailed below.

### Phase 1. Clinician survey: describing current clinical practice

Sixty-five participants with ≥ 2 years working with children with AMC (28 occupational therapists, 37 physical therapists) from nine countries reported on the preferred assessments and interventions used within the areas applicable to their practice. Stretching of upper and lower limbs was the most used intervention. Other frequently used intervention approaches included strengthening, the use of orthotics, positioning, activity-based training, and assistive devices for self-care and mobility. Detailed results are reported by Cachecho and colleagues [20].

### Phase 2. Developing recommendations

Sixteen recommendations on early intervention and motor development, interventions targeting muscle and joint function, orthotics, mobility training and assistive equipment, participation in areas of life (self-care, school, work, leisure, domestic and social), pain management, psychosocial wellbeing, and perioperative rehabilitation were developed. The final version of these recommendations is found in Table 3 and detailed in the Supplementary file 2.

### Phase 3. Consensus building

Forty-one responses were recorded in round 1 and 37 in round 2. Demographic information of participants in both rounds are summarized in Table 2. Of the 41 participants in round 1, seven were individuals with lived experience, 32 were health care professionals and two were researchers. Health care professionals and researchers in round 1 reported they work in hospitals (n = 21), rehabilitation centers (n = 11), private centers (n = 3) and other sites (n = 6) (clinical research department, community school, university, national resource center, motion analysis center), some of them working in more than one setting. Of the 32 health care professionals, 27 had over 10 years of experience. Distribution in round 2 was similar.

**Table 2:** Participants of phase 3. Consensus building

Participants	Round 1 (n = 41)	Round 2 (n = 37)
*Expertise*		
Occupational therapist	11	11
Physical therapist	10	9
Kinesiologist	1	1
Social worker	1	1
Orthopedic surgeon	6	5
Plastic surgeon	1	1
Physiatrist	1	1
Pediatric neurologist	1	1
Lived experience	7	7
Researcher	2	–
*Country*		
USA	17	16
Canada	11	9
Spain	4	3
Israel	2	2
Sweden	2	2
Australia	1	1
France	1	1
Norway	1	1
Poland	1	1
United Kingdom	1	1

Consensus on the 16 recommendations was achieved after two Delphi rounds. All recommendations achieved high level of agreement (mean = 96.6%) in round 1. However, due to the large variability in the level of agreement for seven of the recommendations and based on the comments provided, these were modified and sent for a second round of validation. Table 3 lists the final 16 recommendations, the percent agreement obtained, and the supporting evidence considered to develop the recommendations with their level of evidence. Additional information providing further important details for each set of recommendations, as well as a summary of the studies considered, are provided in a supplementary document (Supplementary file 2). Table 4 provides a summary of rehabilitation goals based on developmental stage.

**Table 3:** Consensus-based recommendations with corresponding agreement and studies considered

Recommendation	Delphi Agreement (%)	Levels of evidence of the studies considered (n)	Review article (reference number)	Indirect evidence (reference number)
*Early intervention and motor development*				
Recommendation 1*.* For children with AMC, starting at birth and during the first year of life, we suggest regular stretching and positioning in conjunction with caregiver education, a home exercise program and orthotics to maximize the window of opportunity to increase passive and active joint range of motion and decrease joint contractures	98.6	Level IV: 6 [27–32]	[33–43]	[44]
Recommendation 2. For children with AMC, in the first 3 years of life, we suggest using developmental stimulation, positioning, and trunk and limb strengthening to optimize motor skills development and tailor strategies to the child’s capacities with assistive devices and/or compensatory strategies as indicated	98.8	Level IV: 6 [27–32]	[33–43]	[44]
*Interventions targeting muscle and joint function*				
Recommendation 3. For children with AMC, after the age of 1 year, we suggest to continue regular stretching, strengthening, positioning, in conjunction with caregiver education, a home exercise program and orthotics throughout growth, to maintain gains and maximize function, joint ROM and alignment, body symmetry, muscle strength, and development	98.8	Level III: 1 [45] Level IV: 4 [46–49]	[43]	[50–55]
Recommendation 4. For children with AMC, we suggest strengthening available muscle groups to increase active range of motion, strength, mobility, stability, and improve overall health	93.1*	Level III: 1 [45] Level IV: 4 [46–49]	[43]	[50–55]
*Orthotics*				
Recommendation 5. For children with AMC, we suggest using orthotics for the upper and/or lower limbs starting in the first year of life and during the life span to improve joint positioning, improve and maintain range of motion, provide joint alignment and stability for standing, walking, and other functional tasks, and maintain correction post-surgery or post- serial casting	95.8*	Level III: 1 [56] Level IV: 17 [27, 29–32, 49, 57–67]	[33, 38]	[68, 69]
Recommendation 6. For children with AMC, exoskeletons for the upper limb may be used to increase function, but there is insufficient evidence to support or reject their use for upper limbs at the current time	89.2*	Level III: 1 [56] Level IV: 17 [27, 29–32, 49, 57–67]	[33, 38]	[68, 69]
*Mobility training and assistive equipment*				
Recommendation 7. For children with AMC, we suggest early mobility training, including use of mobility aids and orthotics as needed, to maximize mobility (e.g., floor mobility, standing, transferring, walking, assisted walking or wheeled mobility) within their environment based on the child’s age and functional needs	99.1	Level IV: 3 [27, 32, 70]	[43, 61, 71, 72]	–
*Participation in areas of life (self-care, school, work, leisure, domestic and social)*				
Recommendation 8. For children with AMC, we suggest maximizing autonomy in self-care activities (feeding, dressing, grooming, toileting, bathing) and other meaningful activities in diverse environments (home, school, work, community) by using a team approach and goal oriented activity-based training tailored to the child’s age and needs, including practice of different strategies, trial of assistive equipment and learning from peers	97.4*	Level IV: 6 [27, 57, 63, 70, 73, 74]	[43]	[75, 76]
Recommendation 9. For children with AMC, we suggest maximizing participation in meaningful activities (school, domestic, leisure and social activities, and work) in diverse environments (home, school, work, community) by supporting accessibility and integration through environmental modifications and advocacy	99.0	Level IV: 6 [27, 57, 63, 70, 73, 74]	[43]	[75, 76]
Recommendation 10. For children with AMC, we suggest providing opportunities for participation in meaningful activities (school, domestic, leisure and social activities, and work) in diverse environments (home, school, work, community) by guiding families in accessing appropriate external resources	98.8	Level IV: 6 [27, 57, 63, 70, 73, 74]	[43]	[75, 76]
*Pain management*				
Recommendation 11. For children with AMC, we suggest evaluating the presence (location, intensity) and type of pain, and its impact on function, in order to tailor the pain management plan	98.8	Level IV: 1 [27]	–	[77]
Recommendation 12. For children with AMC we suggest providing treatment approaches (e.g. soft tissue management, thermal modalities, positioning, energy conservation), orthoses or mobility aids (e.g., walking aids, wheelchair), and/or a home exercise program, based on the child’s needs and tolerance, in order to reduce and/or manage pain	94.8	Level IV: 1 [27]	-	[77]
Recommendation 13. For children with AMC, we suggest offering structured education on the concept of pain and pain management, encouraging self-management strategies, participation in support groups, and facilitating peer-to-peer support, in order to recognize, manage and/or reduce pain	94.8*	Level IV: 1 [27]	-	[77]
*Psychosocial wellbeing*				
Recommendation 14. For children with AMC, to improve psychosocial wellbeing, we suggest using coping strategies, peer-to-peer support and guidance on available resources, based on individual characteristics and contextual circumstances	92.9*	Level IV: 2 [62, 70]	–	[75, 78–81]
*Perioperative rehabilitation*				
Recommendation 15. Pre-operative rehabilitation. For children with AMC undergoing upper or lower limb surgery, we suggest pre-operative rehabilitation, including education, equipment provision, home environment modification, combined with other interventions as needed (in person rehabilitation treatment, home exercise program, psychosocial support), to prepare the family and child for surgery and optimize the child’s joint ROM and strength	96.2*	Level III: 1 [45] Level IV: 3 [48, 82, 83]	–	[84–96]
Recommendation 16. Post-operative rehabilitation. For children with AMC undergoing upper or lower extremity surgery, we suggest implementing rehabilitation interventions targeting muscle and joint function (ROM, stretching, orthotics, and strengthening), and activities (activity training, standing, transferring, walking, recreational activities) and offering psychosocial support when needed, to maximize functional outcomes	99.1	Level III: 2 [45, 97] Level IV: 20 [46–48, 82, 83, 98–112]	–	[113]

*Consensus at Round 2

**Table 4:** Rehabilitation goals based on developmental stage

	Infant	Toddler	School age	Teenager/adult
Therapy	Multiple environments	Multiple environments	School environment	Multiple environments
	High intensity	High intensity	Episodic care	Needs-based services
				Episodic care
Body function	A/PROM	A/PROM	A/PROM	A/PROM and strengthening through activities
	Activation of movements	Activation of movements	Strengthening	Healthy lifestyle-exercise, cardiovascular, minimizing weight gain
	Strengthening	Strengthening	Orthotics	Pain management
	Orthotics/taping	Orthotics/taping	Pain management	Post-operative rehabilitation
	Casting	Casting	Post-operative rehabilitation	
	Pain management	Pain management		
	Post-operative rehabilitation	Post-operative rehabilitation		
Activity/participation	Floor	Mobility-transitional movements; standing/gait; wheeled mobility	Mobility-school accessibility	Mobility-full accessibility within environment/community
	Mobility-transitional movements; rolling, sitting	Fine motor-explorative play	Transfers	
	Fine motor-exploratory play, reaching, grasping, holding	ADL-emerging acquisition	Fine motor-school related	Fine motor-job related
		Emerging community participation	Assistive technology	Assistive technology
			ADL-autonomy from home caregiver	Community participation-education, leisure and social activities, work
			Increased community participation	Transition to adult life
Personal factors	Psychosocial wellbeing-caregivers/family	Psychosocial wellbeing- child, caregivers/family	Psychosocial wellbeing- child, caregivers/family	Psychosocial wellbeing- youth, caregivers/family

Adapted from Wagner and colleagues [43]

*A/PROM* active/passive range of motion, *ADL* activities of daily living

### Phase 4. External appraisal

The five experts were from Brazil (n = 2; Occupational Therapy/Rehabilitation Sciences and Physical Therapy/Pediatrics), Canada (n = 1; Knowledge Translation) and the United States (n = 2; Occupational Therapy and lived experience), and had between 12 and 28 years of experience in these fields. As per the external review using the AGREE-II tool, the overall quality of the rehabilitation recommendations was rated at 96.6%, ranging from 82.5% (Domain 5, applicability) to 97.8% (Domain 1, score and purpose). Only domain 5 scored below 92.1%. One expert scored two out of the 23 items below 3 out of 7; item 14 “*procedure for updating the guideline is provided*” (Domain 3, rigour of development) was scored a 2, and item 21 “the guideline presents monitoring and/or auditing criteria” (Domain 5, applicability) was scored 1. The research team considered the comments provided from the external appraisers and incorporated all the suggestions to ensure that the required procedures would apply.

### Phase 5. Facilitators and barriers to uptake of recommendations

Rehabilitation professionals reported that training, regular team encounters, and a knowledge broker could support the implementation of the recommendations in practice. Other facilitators included the collaboration of managers and professionals in the development of the recommendations and the presentation of the recommendations in a visually appealing and easily accessible format. Some barriers included lack of time and clinical resources and difficulty adapting the recommendations. Detailed results of this phase are reported in a recent publication by McBain and colleagues [26].

## Discussion

This multiphase project embedded in an iKT approach led to the development of 16 consensus-based recommendations for the rehabilitation management of children with AMC. Clinicians across several disciplines, individuals with lived experience and their families from 10 countries contributed to this effort. Similar to other rare disorders such as juvenile dermatomyositis [114] and osteogenesis imperfecta [115], we used an evidence-informed consensus process with experts across several countries to develop recommendations. More recently, individuals with lived experience in spinal muscular atrophy have also been involved in the development of consensus-based recommendations [116]. The use of the GRADE approach allowed consideration of the experience and expertise of the panel members, their values and preferences, and ascertained stakeholder’s perspectives about the advantages and disadvantages of proposed interventions while considering the available research evidence.

In order to promote the adoption of the consensus-based recommendations widely, the 16 recommendations will be translated and forwarded to patient advocacy groups as proposed by Mercuri and colleagues [116], and to rehabilitation practitioners and other potential end-users, using professional networks and publications, conferences, the internet and social media channels. Implementation of clinical recommendations, monitoring and evaluation, are essential to promote their use. Future studies should evaluate the effectiveness of the proposed recommendations to improve service delivery and patient health using appropriate designs, while considering perceived facilitators and barriers to guideline uptake in rehabilitation [26]. To this end, online learning modules pertaining to the content of the rehabilitation recommendations will be pilot-tested using a multisite cluster randomized controlled trial measuring service and patient health outcomes.

Although there is limited or low quality evidence regarding rehabilitation treatment effectiveness of individuals with AMC, the use of the AGREE II ensured a rigorous evaluation process of the consensus-based recommendations. Therefore, the application of the AGREE II using external experts in rehabilitation, knowledge translation and lived experience ensured that the development process was transparent, well documented and replicable. While low-quality evidence may not provide strong support for recommendations, the methodology used including knowledge synthesis, consensus-based methodology and the GRADE approach supports the rigor and relevance of the project. Limitations of this project include the representation from only two low-middle income countries (i.e., Peru, Poland). The applicability of the recommendations may be limited by regional, cultural, and socio-economic factors. The overall quality of the evidence of the rehabilitation interventions with children with AMC is weak at this time, and the strength of the recommendations is low, implying that desirable effects probably outweigh the undesirable effects, but appreciable uncertainty exists. This work highlights the need for more robust evidence in the field of rehabilitation in AMC. Multicenter collaborations can enable the design of rigorous studies with large samples to produce stronger evidence in this field. Further, knowledge translation products for rare diseases such as the Rare Knowledge Mining Methodological Framework that includes other sources of evidence such as registry information, qualitative studies as well as the involvement of expert patients may be considered [117]. As research evidence continues to grow, the recommendations will be updated over time while considering topics that were not or only briefly addressed in this version. Such topics may include, but are not limited to, the role of rehabilitation in prenatal counselling and in the transition to adulthood, including aspects of intimacy and relationships. Finally, these recommendations pertain to rehabilitation practices specifically. Recommendations to guide best practice for the multidisciplinary management of AMC (i.e., prenatal, medical, genetic, nursing, etc.) would complement this project and should be considered in future work.

## Conclusions

The development of the consensus-based recommendations for the rehabilitation of children with AMC used an iKT approach and involved clinicians from several health disciplines, researchers, and people with lived experience across 10 countries. In total, 16 recommendations were formulated to guide clinical decision-making. Due to the limited research evidence pertaining to rehabilitation in AMC, the expertise of clinicians and individuals with lived experience was key to this endeavor. This highlights the need for more empirical evidence through multicentric prospective studies evaluating outcomes of specific rehabilitation interventions. Results from this study will be formally disseminated to healthcare users and providers to promote implementation of the recommendations in practice by addressing identified facilitators and barriers to implementation, thereby contributing to child- and family-centered care in AMC.

## Supplementary Information

Additional file 1.

Additional file 2.

## Data Availability

The data supporting the recommendations are available in this paper, the Supplementary file 1 and in the original reference describing the outcomes of phase 1 (clinician survey) 10.1080/09638288.2022.2161644.

## Abbreviations

A/PROM: Active/passive range of motion
ADL: Activities of daily living
AGREE II: Appraisal of Guidelines for Research and Evaluation II
AMC: Arthrogryposis multiplex congenita
GRADE: Grading of recommendations, assessment, development and evaluations framework
iKT: Integrated knowledge translation
OT: Occupational therapist
PT: Physical therapist
TDF: Theoretical domains framework
