# Supplementary material for: Consensus-based recommendations for the rehabilitation of children with arthrogryposis multiplex congenita: an integrated knowledge translation approach
Source: Orphanet J Rare Dis. 2025 Apr 9;20:168. doi: 10.1186/s13023-025-03671-x (PMC11983950; doi:10.1186/s13023-025-03671-x)
Supplement: Supplementary file 1 — Additional file 1. [file 13023_2025_3671_MOESM1_ESM.docx]

Studies reporting on rehabilitation approaches used with children with AMC

| **Study** | **Type of treatment** | **Study design** | **Population (age)** | **Intervention** | **Comparison** | **Outcome** | **Timing** |
| --- | --- | --- | --- | --- | --- | --- | --- |
| Azbell & Dannemiller, 2015 | Stretching positioning strengthening splinting/ orthotics parental education | Case study | Infant with Amyoplasia (n=1; 11days) | Weekly PT/OT sessions including stretching, positioning, strengthening and splinting. | None | Improvement in patient satisfaction scores, prone tolerance, range of motion (neck, UE, LE), reaching and grasping. Improved hand to midlines but not to mouth. PDMS2 gross motor scores below average for locomotion. Remaining ROM limitations affecting gross motor skills. Pain measurement (FLACC) was high during PROM in the first days of life, decreased after a few minutes of ROM and decreased over the following months. | Starting at 11 days to 9 months |
| Babik et al., 2016 | Exoskeleton (WREX) | Case study | Infant with Amyoplasia (n=1, 8months) | WREX (60 min daily) + OT/PT 1h/week. | None | No change in ROM but improvement in against gravity and gravity-eliminated movement, improvement in reaching ability (low level) and time spent in contact with objects. Improvements increased over time throughout duration of intervention. | Starting at 8 month old, for 3 months |
| Babik, Cunha & Lobo, 2019 | Exoskeleton (Playskin Lift^TM^) | ABA single-subject design | Children with AMC (n=17, Average 13.9 ± 8.7 months) | Playskin used for structured intervention activities (30–45 minutes daily) to encourage children to reach for objects placed at different heights and to promote free play | None | While wearing the Playskin: significant improvement in lifting toys from the surface and in contacting toys with both hands (at a low level). Without the Playskin: performance of many behaviors showed improvements. Improved shoulder AROM against gravity. | - |
| Bartonek, Eriksson & Gutierrez‐Farewik, 2007 | Orthotics | Case series | children/adolescents with motor disorders (n=17, 4 with AMC, average 11 years and 11 months) | carbon fiber spring orthoses (CFSO) | AFO/KAFO | Improved ankle plantarflexion moment, ankle positive work, and stride length. The CFSO did not suit all participants based on parent questionnaire. | For 2-3 weeks |
| Binkiewicz-Glinska et al., 2013 | Stretching, joint mobilization, kinesiotaping, development stimulation | Case report | Infant with severe AMC and perinatal hypoxia (n=1; 0-26 weeks) | Rehabilitation (stretching, articular mobilization, corrective kinesiotaping) + development stimulation (positional therapy, stimulation) + orthopaedic treatments (manipulative plasters) | None | Improvement in ROM, as well as in the function of the child | Starting at birth, for 26 weeks |
| Dalton & Hoyt-Hallet, 2013 | Assistive technology | Case report | Child with Amyoplasia (n=1, 12 years) | wheelchair adaptations (assistive technology): soft ball handle joystick, Micro Lever Switch®, VMax® communication device, Picture WordPower® communication software, Daessy RigidMount® and Invacare Quantum 610® power wheelchair | None | Improved academic performance, communication and better ability to initiate conversations and converse about any topic | - |
| Eriksson et al., 2015 | Orthotics | Descriptive study | Children with AMC (n=37; average 9.7 years) | AMC 1 (5 children) used KAFOs with locked knee joints, AMC 2 (10 children) used KAFOs with open knee joints or AFOs, and AMC 3 (11 children) used shoes. | Typically developing children | All AMC groups showed less hip extension than the control group, but hip flexion moment was significantly lower only in AMC 1 AMC 1, who had weak knee extensors, were helped by their locked KAFOs and showed similar knee extension moment as the other groups. With adequate orthotic support, children with AMC and even with severe weakness and contractures can achieve walking. | - |
| Eriksson et al., 2018 | Orthotics | Cross-sectional Study | AMC (n=33; average 10.5 years) | Lower limb orthosis (KAFO, AFO) (Ort-D group). 14/18 used them more than 8h a day, 2/18 between 5 and 8h a day and 2/18 less than 5h a day. | No orthosis (Non-Ort group) | Children with AMC had significantly lower Child Health Questionnaire (CHQ-PF50) scores in 9 of 12 subscales compared to healthy controls. No difference found in the psychosocial summary score between the AMC group and the control on the CHQ-PF50. Lower scores found in AMC compared to the control for: “parent impact/emotional”, “self-esteem” and “behavior”. The children’s reported perceived health with EQ-5D youth did not show any difference between children using orthoses or children using only shoes. Pediatric Evaluation of Disability Inventory showed less mobility in Ort-D than in Non-Ort. In total, participants were ‘quite satisfied’ with their orthoses. | - |
| Gür et al., 2016 | Serial splinting/orthotics exercises activity training | Pilot study | Children with AMC (n=2; 6 and 7 months) | Serial splinting for bilateral knee flexion contractures. A static knee orthosis was used for 15-18h/day, renewed every 15 to 30-days. Terminated if no change in knee flexion contracture after two consecutive measurements. Physiotherapy including exercises and activity training | None | Improvement in bilateral passive extension in both cases (increase of 40° and 25°). | Follow-up duration 1 year |
| Hall & Hammock, 1979 | Assistive devices | Case study | Children with AMC (n=2; 8 years and teenage girl) | Personalized simple feeding device & toileting ramp  - Feeding device: enables the child to use a spoon or fork by head control.  -Details /plans of the toileting ramp and its construction are available from the authors upon request. | None | Improved ability to self-feed and transfer on toilet. | - |
| Haumont et al., 2011 | Exoskeleton (WREX) | Prospective case control study | Children with muscle weakness (n=3), only 1 child with AMC (2 years) | -WREX fixed to a back brace as the patient was ambulatory   -no specific intervention provided but used in various daily activities. | None | Motion analysis showed a marked improvement in UE function with the WREX. Child with AMC showed improved self-feeding abilities in different environments. Helps alleviate UE fatigue. | - |
| Kamil & Correia, 1990 | Stretching and splinting | Case report | Infant with AMC with severe elbow extension contracture (n=1, 7 days to 12 months) | Gentle stretching and serial splinting for 12 hours a day Bivalve long arm elbow flexion splint until 8 months. Dynamic elbow flexion splint at 9months worn at home for two 1.5 hour periods | None | Improved elbow passive flexion from 20deg to 100 deg at 12months. Improved hand to mouth reach for self-feeding with splint on. | Starting at 7 days of age to 12 months |
| Lobo et al., 2016 | Exoskeleton (Playskin Lift^TM^) | Case report | Toddler with AMC (n=1, 23 months) | Exoskeletal garment (Playskin lift) | None | Improved ability to reach for and contact toys at chest and eye level, and to play on the floor, while wearing the exoskeleton. Most of toy contact involved toys that are resting on body or surface, but also demonstrated some unsupported contact while wearing the garment. More time interacting with multiple toys for stacking, banging, and putting toys in and out of one another when wearing the device than without it. | - |
| Moore et al., 1990 | Orthotics strengthening mobilization | Case study | Child with AMC (n=1; 9 years) | HKAFOs articulated at hips and knees, sections reinforced with steel side members, gas spring applied across hip and knee to provide extending moments, worn 20min/day, gradually increasing to 3h/day (evenings). Continued wear of KAFOs during the day + physiotherapy (strengthening and mobilization exercises). | None | After 10 months: Hip and knee contractures reduced, gait improved, knee extension improved but some flexion lost without impact on activities. Orthotic control was stronger than the exercise regimen. After 18 months, child still using orthosis every evening and improvements in joint ranges have been maintained. | Follow-up duration 18 months |
| Palmer et al., 1985 | Stretching and splinting | Retrospective case series | Children with AMC (n=95; age not clearly specified) | Early and intensive A/PROM. Daily passive stretching + serial splinting. Home program of stretching completed 4x/day. Weekly follow-up by therapist and/or orthopedic surgeon. Various body areas targeted. | None | Those who underwent this program required less surgery afterwards. The earlier the program was initiated the better were results. Improvement in ROM to variable degrees in the different body areas and improvement in function. | Most seen before 3 months of age |
| Pritchard-Wiart et al., 2019 | Early assisted mobility (toy car) | Mixed method case series | Child with AMC and hypotonia (n=1, 13 months) | early assisted mobility (toy car) 2.4 times per week for 30-120min. | None | Increased curiosity and wanting to explore, increased play, increased communication, motivation in general, can keep up with siblings and had more independent mobility. | For 5 weeks |
| Rahman et al., 2006 | Exoskeleton (WREX) | Case series | Children with AMC (n=5; 6-14 years) | Training provided for 2-3 sessions, no details in paper | None | Continued use by 4/5 participants. One has negative comments from peers and stopped using it. WREX improved participant's ability to self-feed, write and raise hand in school. It is used at home and in school. | 5-22 months of use |
| Sala, Rosenthal & Grant, 1996 | PROM, splinting, developmental activities, parent education | Case study | Infant Amyoplasia (n=1, 4 months) | Outpatient PT OT including parental instruction, PROM, Facilitation of AROM, Positioning equipment, splinting, developmental activities. LE serial casting. | None | Improvements in AROM (neck, UE) and PROM (neck, UE, LE), and in the ability to move the UE when presented with a toy in an attempt to approach it. Participant still unable to bring her hands to midline, grasp or swipe at a toy. Improved adapted and independent seating. | - |
| Smith & Drennan, 2002 | Casting, stretching, splinting | case study | Infants with distal (n=5) and classic arthrogyrposis (n=12) average age 6 months. | Passive stretching, serial casting and custom wrist orthotics. Average number of casting sessions per patient/extremity: 5; with an average length of 56 days per session. All patients with distal arthrogryposis required a single casting session to gain significant passive wrist extension. Most patients with classic arthrogryposis underwent four or more casting sessions. | None | Patients with distal arthrogryposis had the largest improvement in passive wrist motion, were more functionally independent at final follow-up, and had no recurrence of deformity. Patients with classic arthrogryposis had rigid wrist flexion contractures and a 75% incidence of deformity recurrence after casting. At final follow-up, these patients remained functionally dependent, requiring >50% assistance with activities of daily living, and had less improvement in wrist motion. | Follow-up duration 1-16 yrs (mean 6 yrs) |
| Tsuyuguchi et al., 1985 | Splinting or PROM | Case series | AMC (n=43; age not clearly indicated) | Forty-two hands were treated with splinting alone, and 16 hands with surgery. 17 hands were followed conservatively without splinting or surgery (with passive range of motion exercises). | None | Patients showed good response to splinting.10 patients (16 hands) who had severe deformity or no response to splinting were treated surgically. Satisfactory results were obtained in 12 of 16 hands. In the 17 hands followed conservatively without splinting, the condition completely resolved in five hands within 6 months; however, 12 hands showed slight remission or no change. | Variable according to clinical presentation |
| Wee et al., 2019 | Spring powered elbow orthosis | Case study | Teenager with AMC (n=1, 18 years) | The prototype device used was a lightweight, spring-powered flexion orthosis. No information on training provided. | None | Improvement of elbow flexion from 87deg without the brace to 120 with the brace. It allowed participant to bring hand close to mouth without using the other hand for assistance. | - |

**AMC**: arthrogryposis multiplex congenital; **UE**: Upper extremity; **LE**: Lower extremity; **PDMS2**: Peabody Developmental Motor Scales 2; **ROM**: range of motion; **FLACC**: Face, Legs, Activity, Cry, and Consolability; **AROM**: active range of motion; **PROM**: passive range of motion; **PT**: physiotherapy; **OT**: occupational therapy; **AFO**: ankle-foot-orthosis; **KAFO**: knee-ankle-foot-orthosis;

Studies reporting on rehabilitation approaches used with children with AMC in a perioperative context

| **Study** | | **Type of treatment** | **Study design** | **Population (age)** | **Intervention** | **Comparison** | **Outcome** | **Timing** |
| --- | --- | --- | --- | --- | --- | --- | --- | --- |
| Asif et al., 2004 | Splinting ROM ambulation | | Case study | Infants with AMC (n=4, average 23months | open reduction surgery +/- bony procedure, followed by hip spica (3months), night splinting (3months) and gradual ROM and ambulation | None | Improvement in Tonnis grading system, acetabular index, hip ROM | Follow-up duration average 4 years |
| Aydin et al., 2016 | PT | | Case study | Infants with AMC and hip dislocation (n=7; average 3.5months) | Open reduction + spica cast (3months) + PT for hip exercises to prevent stiffness (not detailed) | None | Improved ROM and hip stability. However, 4 patients needed additional hip surgery because of recurrence | - |
| Bennett et al., 1985 | | Pre-op: AROM, PROM, night time splinting  post-op: splinting | Case series | Children AMC [UE deformities] (n=36, age not available) | -10 patients has early PT/OT treatment A/PROM exercises + overnight orthoses (0 -2 years of age), -1 patient had severe deformities, no active treatment  -25 underwent 56 operative procedures to improve upper extremity function (at 5-6 years) following by splinting. | None | Surgical intervention results were satisfactory and improved function in 75% of the patients  Early and postoperative splinting until skeletal maturity prevented the recurrence of wrist deformities | Followed different patients at different age stages for 20 years |
| Carroll & Hill, 1970 | Splinting and AROM | | Case series | Youth and adults with AMC with elbow flexion contractures (n=15, 7-33 years) | Triceps transfer procedure to restore elbow flexion  _Splint after surgery for 4 weeks for immobilization  _active flexion exercises at 4 weeks | None | Some patients showed improved elbow flexion and others did not. | - |
| Chomiak, Dungl & Vcelák, 2014 | Electrical stimulation, AROM, PROM, strengthening. | | Case series | Children with classic arthrogryposis (n=9, average 6.3 years) | Pectoralis major transfer to restore active elbow flexion.  Post-op rehab: transcutaneous electric stimulation of transferred muscle, elbow and shoulder exercises with isotonic concentric contraction in the first phase (4 to 12 weeks). In the second phase, A/PROM of the elbow for a minimum of 15 minutes daily for 3 months. | None | Significant improvement of function for daily living activities in 5 extremities. Four extremities remained unsatisfactory. Significant elbow flexion contracture developed in 4 extremities. | Follow-up duration 13-16 years |
| Church et al., 2020 | Orthotics | | Retrospective review | idiopathic clubfoot (n=89), arthrogryposis (n=28); 4.8 ± 0.8 years) | Ponseti method +/- percutaneous Achilles tenotomy. After the final cast, Ponseti braces were worn for 3 months for 23h/day and then overnight and during naps until the age of 5 years. Children with clubfoot associated with arthrogryposis also wore AFOs daily. Those who could not tolerate the Ponseti braces at night wore their AFOs. | Typically developing children (n=72) | Residual equinovarus deformity and ankle ROM limitations in both the arthrogryposis and clubfoot group compared to typically developing peers. Significantly, more limited gross motor function in the arthrogryposis group as per GMFM and PODCI + presence of pain, compared to the idiopathic clubfoot group. The arthrogryposis group and typically developing group differed in the upper extremity and physical function, transfer and basic mobility, sports, and global function. | - |
| Dangles & Bilos, 1981 | Splinting and AROM | | Case series | Children with AMC with thumb adduction contracture (n=4, average 5 years) | Surgical correction of thumb deformities and postoperative splints, removed intermittently for training of the opponensplasty by OT. Night time splinting for 6 months. | None | An increased thumb web space was achieved and maintained.  In 3/4 patients an ability to activate opponensplasty was developed  A stable MP joint was achieved in 3/4 patients.  Effectiveness of the splint was not directly addressed | - |
| Frizzell, Kozin & Zlotolow, 2020 | Splinting, ROM, strengthening | | Retrospective Chart Review | Children with Amyoplasia (n=5, 6 upper extremities, Average: 7.8 ± 3 years) | Bipolar Latissimus Dorsi Transfer followed by a posterior long-arm orthosis with elbow at 100-110, for 4 to 6 weeks. Then therapy started and locking hinged elbow brace at 90 degrees used. Elbow extension was increased in 15-degree increments weekly until full extension was allowed. Resistance and weight bearing were allowed at 3 months after surgery. | None | Right after surgery, passive elbow flexion at least 90deg, but no active elbow flexion. At latest follow-up, mean active arc of motion was 76 +/- 14deg and active elbow flexion was 108 +/- 15deg.  Good to great satisfaction at 10months follow-up. | Average follow-up duration 30.2 months |
| Fucs et al., 2005 | Orthotics/ splinting strengthening ROM | | Retrospective case series | Children Amyoplsia (n=8; average 2 years 8 months) | Quadricepsplasty + cast change every 3-4 days alternating flexion and extension for 2weeks. At 3 months KAFO with articulated knee to initiate walking. During the day alternated knee extension vs flexion for walking vs sitting + physiotherapy (ROM and strengthening). At night splinting in flexion for 12 weeks. Daytime functional brace depended on the need. | None | Improved knee PROM (flexion and extension). Functionally at the final evaluation, seven patients were community ambulators: two with KAFOs, and one household walker with a KAFO. | Follow-up duration 11 years and 2 months |
| Hageman et al., 2019 | AROM, PROM | | Case series | Children with AMC (n=4, 6 upper extremities; 3-6 months) | Nerve transfer from the median to musculocutaneous nerve. Active and passive exercises and physiotherapy were initiated in all patients 1 week after surgery and follow up took place at 6 and 12 months after surgery and later annually. | None | 2/5 extremities reached elbow flexion grade M4, 2/5 M3 and 1/5 M1.  1 patient had botox injection in triceps in addition to the transfer. | - |
| Ho & Karol, 2008 | Orthotics | | Retrospective case study | Children with AMC (n=32, 50 knee releases; average 2.7 years. | Knee releases + 30/50 of knees were braced in KAFOs, 13/50 of knees were placed in HKAFOs. | None | Improvement in knee flexion ROM. At final follow-up, 11/50 of knees used KAFOs, and 1/50 used HKAFO. | Follow-up duration average 11.9 years |
| Leonchuk et al., 2020 | ROM, stretching, orthotics, swimming, strengthening, wax therapy and electrical stimulation | | Retrospective case series | MCC, popliteal pterygium syndrome, complete tibial hemimelia (n=8, 16 knees; average 4 years) | Ilizarov application followed by long-leg walking casts with knee in full extension for 2 months, followed by orthoses and supports + in-patient PT 4x/days for 10 days (wax therapy and quadriceps electric stimulation +ROM and stretching). Home program including strengthening and balancing exercises. Swimming was encouraged wherever feasible. | None | Improvement in knee ROM. All participants were non ambulatory became ambulatory with brace and support. | 2 year follow-up |
| Moghadam et al., 2015 | Pre-op: manipulation casting  Post-op: ROM, strengthening Home exercice at home | | Prospective study | Amyoplasia (n=14, 23 knees, average 18 months) | No response to manipulation and serial casting pre-op. Quadriceplasty followed by knees long-leg cast for 3 weeks. After cast removal, PT (ROM and strengthening) for 1 months then home program for at least 3 months. | None | Significant improvement in knee flexion and in mean ambulation score (FMS). | - |
| Niki, Staheli & Mosca, 1997 | Pre-op: casting  Post-op: orthotics | | Case study | Children with Amyoplasia (n=22, 41 feet; average 1.8months | Pre-op: serial casting. Posteromedial-lateral release (PMLR) followed by casting for average of 6 weeks (4-12), changed every 1.8weeks. Then splinting at night, bracing during the day.  Secondary operative procedures to correct recurrent deformity were performed in 20 feet. | None | After PMR, correction of deformity without recurrence was achieved in 11 (27%). Recurrent deformity of varying degrees occurred in 30 (73%). No significant difference in the duration of postoperative cast immobilization was found in the 30 feet with recurrent deformity (6.1 ± 1.5 weeks) compared with those 11 feet without recurrent deformity (6.9 ± 1.9 weeks). Long-term use of splinting at night after the primary PMR helped to prevent recurrence of deformity. | - |
| Ramirez et al., 2017 | Splinting and ROM | | Retrospective chart review | Children with arthrogryposis (n=38; 2 groups: average ages 3.3 and 5.7 years) | -Release group: Patients had only a posterior elbow release + orthosis wear for 2 to 3 weeks. Simultaneous group: posterior elbow release + humeral rotational osteotomy. Long-arm cast immobilization was 4 to 6 weeks for a single plate or pins and 2 to 3 weeks for double plates. -Following immobilization, patients in both groups had a ROM protocol. If more therapy was needed to maintain motion, patients were supplemented with alternating flexion and extension orthoses as warranted for a minimum of 2 months. |  | Increase in total arc of motion in both groups by increasing flexion.  Both groups lost some elbow extension  At all stages of follow-up (early, 1-2 years and final), the release group had a significantly larger arc of motion and a smaller flexion contracture compared to the other group | Release group: average follow-up duration 44.5years  Simultaneous group: average follow-up duration 39.8years |
| Schwering, 2015 | pre-op: casting, PT, stretching  post-op: parental education, PT, OT, splinting, orthoses, strengthening | | Retrospective case series | Youths with arthrogryposis, spina bifida, or CP (n=45, 10 with AMC, average 4 years 6 months | Pre-op: casting and manual stretching. Achilles tendon lengthening followed by PT/OT at 5 weeks post-op + orthosis use. Teaching of stimulation treatment to parents (stroking of inner border and sole of foot) to be done 3x/days for 15 min to strengthen supinator and plantar flexors. . | None | The assessment of the results of the interventions was based on the Walker Score (function, clinical appearance, range of motion, and radiographic findings). The patients with AMC had an improvement from 3.6 (preoperative) to 9.9 on the walker score at follow up. | Follow-up duration average 7 years and 3 months |
| Sochol, Edwards III & Stevanovic, 2020 | pre-op: PROM  post-op: splinting, therapy, muscle stimulator | | Case study | Child with arthgroyposis (n=1; 5 years) | Pre-op: over 1 year of OT to achieve maximum elbow joint motion, reached a plateau  Surgery: Free Functional Gracilis Muscle Transfer Post-op: long-arm posterior splint at 90° of elbow flexion with neutral rotation. Hand therapy at 8 weeks postoperatively, and a muscle stimulator device was used during therapy sessions. | None | Achieved muscle grade M4 (compared to 0 pre-op) and arc of active movement of the elbow of 25 to 140deg.  Improvement was gradual over the first year. Improvement in ADLs and patient/parent satisfaction. | - |
| Szöke et al., 1996 | Post-op: orthotics spontaneous activity | | Case series | Children with Amyoplasia (n=95; 8.9months | Open reduction of hip dislocation followed by spica cast between 5 to 8 weeks (mean 5.9 weeks). After the spica cast immobilization, Seattle abduction orthosis was used on 20 hips with spontaneous activity. | None | Satisfactory acetabular development. 80% rated good, 12% fair, 8% poor. Complications included one early re-dislocation, two hips with stiffness, and 4/25 hips with avascular necrosis. | - |
| Van bosse et al., 2007 | Orthotics ROM PT gait training | | Case study | AMC (n=7, 10 knees; average 7.3 years) | Posterior knee soft tissue releases and gradual contracture distraction with an Ilizarov external fixator. After frame removal, cast for 2weeks then use of KAFO with locking knee hinges. PT begins with gait training and ROM. KAFO is worn in full extension for 3months and only removed for bathing and PT. Then, it is worn at nighttime and day time as needed for ambulation. | None | All knees were corrected to full extension. All patients improved their ambulation capacity. | Average follow-up duration 4.4 years |
| Van Heest et al., 2008 | PROM, AROM, splinting | | Retrospective review | Children with Amyoplasia (n=23, 29 elbows; average 2.9 years) | -Surgical procedure: posterior elbow capsulotomy with triceps lengthening + immobilization in 90° of flexion with PROM allowed as soon as the patient tolerated it. Therapy was advanced to include hand-to-mouth activities with passive flexion limited to 90° during the first month to protect the triceps lengthening and then advanced to full passive flexion. -A locking hinge splint was used. If elbow extension loss, nighttime wear was alternated between elbow flexion and extension.  -Use of the splint was discontinued during the day after 406 weeks but was continued at night for six months. | None | Improvement in the arc of motion of all elbow. All children were able to reach the mouth using passive assistance and twenty-two children were able to feed themselves independently. The twenty-one children with less than grade-3 elbow-flexion strength required the use of passive assistance. | - |
| Van Heest, Waters & Simmons, 1998 | AROM, strengthening. | | Case study | children with AMC (n=18, 1-14 years) | Elbow capsulotomies with triceps lengthening +/- tendon transfers + immobilization for 4 weeks using different immobilizations based on tendon transferred. Active use of transfer at 6 weeks, resistive exercises at 8 weeks. Elbow protected in sling until transfer could be controlled. |  | Improved elbow ROM. The 18 tendon transfers results: biceps-triceps: 7 good, 1 fair, and 1 poor; pectoralis-biceps 1 good, 3 fair, and 1 poor; latissimus dorsi-biceps 2 good and 2 fair. Improved function in 13 transfers. Decreased function in 5 due to residual flexion contracture or because surgery was on the non-dominant side. | - |
| Wall et al., 2017 | ROM, splinting | | Retrospective Chart Review | Children with arthrogryposis (n=9; 14 extremities; average 6.5 years) | Distal humerus external rotation osteotomy. At 2 weeks, gentle elbow ROM + sling for the following 6 weeks, except for bathing and exercises. Weight bearing at 2 months from surgery. The postoperative plan was modified based on concomitant surgeries. | None | 1 year post op, improved external rotation in resting posture. Improved hand position (palms facing midline). Handwriting improved in 5 patients. | Average follow-up duration 1.9 years |
| Zargarbashi et al., 2017 | Splinting, AROM, PROM | | Case series | Children with arthrogryposis (n=11, 13 extremities, average 5.69±2.49 years) | -Pedicled lattissimus dorsi bipolar muscle transfer. - Postoperative rehabilitation program: immobilization for 6 weeks with the elbow at 90°, followed by a posterior splint at night for an additional 6 weeks + AROM in PT. Passive elbow extension was permitted as soon as a functional elbow flexion was achieved. | None | Function of 12 elbows graded as excellent or good, and 1 graded as poor. Improved active elbow flexion and strength (M4 grade in 10/11 patients).  Improvement in ADLs in 10/13 limbs. | Average follow-up duration 27.31 +/- 17.8yrs. |

**AMC**: arthrogryposis multiplex congenital; **UE**: Upper extremity; **LE**: Lower extremity; **ROM**: range of motion; **AROM**: active range of motion; **PROM**: passive range of motion; **PT**: physiotherapy; **OT**: occupational therapy; **AFO**: ankle-foot-orthosis; **KAFO**: knee-ankle-foot-orthosis; **GMFM**: Gross Motor Function Measure; **PODCI**: Pediatric Outcomes Data Collection Instrument; **MCC**: multiple congenital contractures; **FMS**: Functional Mobility Scale.
