# Supplementary material for: Consensus-based recommendations for the rehabilitation of children with arthrogryposis multiplex congenita: an integrated knowledge translation approach
Source: Orphanet J Rare Dis. 2025 Apr 9;20:168. doi: 10.1186/s13023-025-03671-x (PMC11983950; doi:10.1186/s13023-025-03671-x)
Supplement: Supplementary file 2 — Additional file 2. [file 13023_2025_3671_MOESM2_ESM.docx]

**Consensus-based recommendations**

This document consists of 16 recommendations on the early intervention and motor development, interventions targeting muscle and joint function, orthotics, mobility training and assistive equipment, participation in areas of life (self-care, school, work, leisure, domestic and social), pain management, psychosocial wellbeing, and perioperative rehabilitation. Additional information, remarks, and studies considered will follow each of the recommendations.

1. **Early intervention and motor development**

**Recommendation 1*.*** For children with AMC, starting at birth and during the first year of life, we suggest regular stretching and positioning in conjunction with caregiver education, a home exercise program and orthotics to maximize the window of opportunity to increase passive and active joint range of motion and decrease joint contractures.

**Recommendation 2.** For children with AMC, in the first 3 years of life, we suggest using developmental stimulation, positioning, and trunk and limb strengthening to optimize motor skills development and tailor strategies to the child’s capacities with assistive devices and/or compensatory strategies as indicated.

*Additional information*

Maximizing joint range of motion: In the first year of life, children with AMC have good potential to improve their range of motion (ROM) and decrease the contractures in their joints; therefore, rehabilitation should start as early as possible. The home exercise program should be performed at least **daily** by family or caregivers (e.g. daycare provider) and **regularly** by clinicians (once or twice per month depending on the context and the need). Stretching may include soft tissue manipulation, serial casting, orthotics or a combination of these modalities in order to improve ROM and decrease contractures over time. Before stretching, the therapist should know what the axis of joint movement is to inform the direction of stretching. Stretching should be gentle and should not cause pain to the child. Stretching with too much strength may cause irritation or damage to the joints, in cases of deformity of the bone epiphysis for example. Prior to starting a stretching program, evaluation of the child by a medical team is important to determine if there are any contraindications. Some contraindications to stretching may include bone fragility as seen with Bruck syndrome, and in cases of joint dislocation. Certain bony deformities or synostoses cannot be treated with stretching. When stretching we recommend 6 repetitions of 30 seconds each, or 3 repetitions of 1 minute each. Duration of stretching can be decreased or increased depending on the child’s level of tolerance. The duration of the stretch can be shorter at the beginning but more frequently repeated, then increased gradually. Stretching can be incorporated during diaper changes, bath time, play or sleep. In addition, after stretching exercises, active movements should be encouraged. Use of orthoses at night in the presence of joint contractures is strongly recommended, to reduce contractures and ensure adequate joint alignment. Other positioning techniques (e.g., positioning in supine or prone with adequate support) should be used as well.

Motor development: In the upper limbs, wrist and finger alignment is important for hand function and elbow ROM for hand to mouth and reaching midline. Interventions to improve joint ROM and to stimulate motor development and hand function (i.e., head control, rolling, sitting, reaching, grasping, hand to mouth, bilateral hand use, etc.) are complementary and should be provided in parallel to achieve functional goals. Improvement in lower limb ROM in the first year of life will help determine the potential for sitting, standing and walking. Strengthening can begin when an active muscle contraction is palpated. When working on strengthening of the trunk and limbs, progress according to the child’s capacities, from gravity eliminated planes to anti- gravity movements, when possible. Support may be required to encourage movement in an antigravity plane. Aquatic therapy, when available, can be used to encourage movement and strengthening. In the presence of spine deformities, collaboration with a spine specialist is recommended. Should torticollis be present, specific positioning and stretching exercises should be provided.

Education and home programs: Caregiver education is key to support caregiver and infant interaction and to ensure exercises are carried out safely and regularly at home and in the different settings (e.g., daycare). Demonstration of handling techniques and practice with the therapists are important to ensure proper understanding and ease with the recommended stretching exercises and home exercise program. Using reference materials such as written instruction or videos may be helpful for caregivers. Children with AMC may not always achieve all expected developmental milestones and these may be reached at different ages. When delivery of a home exercise program is challenging (e.g., socio economic barriers, time constraints, presence of other children at home), consider increasing the frequency of therapies provided by a clinician in the first year of life.

*Studies considered*

Six case reports discussed the use of conservative treatments with infants with AMC. Two case studies on infants with Amyoplasia^1,2^ reported improvement in ROM, joint alignment of the hips, prone tolerance, reaching, grasping, sitting, and weight bearing after using daily stretching, strengthening, positioning, splinting or serial casting, and regular PT and OT. A case series on infants with AMC^3^ using a similar treatment demonstrated improvement in joint motion and therefore fewer operative procedures required, especially for patients that received early and frequent interventions before being treated with any surgical procedures. Another case report^4^ of an infant from birth to 26 weeks of age showed improvement in knee joint flexion and general ROM and function with rehabilitation interventions (e.g., stretching, articular mobilization, corrective kinesiotaping), developmental stimulation, positional therapy, and manipulative casting. A case study on two babies with AMC^5^ showed that the use of serial splinting for the knees over one year improved bilateral knee extension. Similarly, in a case report^6^ on an infant with severe elbow extension contractures, splints were used to effectively accomplish gradual elbow stretching in which the infant demonstrated improvement in ROM. In addition, physiotherapy treatment consisting of exercises and activity training contributed to improve head control in all positions and the ability to cruise. Several review articles have reinforced the importance of early intervention in children with AMC by using the above-mentioned approaches^7-17^. Moreover, indirect evidence from a systematic review concluded that despite limited evidence, early intervention combining developmental stimulation, trial and error, and support of parent-child interaction in infants at high risk of cerebral palsy may be the best approach to promote motor and cognitive development as well as family wellbeing^18^.

1. **Interventions targeting muscle and joint function**

**Recommendation 3**. For children with AMC, after the age of 1 year, we suggest to continue regular stretching, strengthening, positioning, in conjunction with caregiver education, a home exercise program and orthotics, throughout growth, to maintain gains and maximize function, joint ROM and alignment, body symmetry, muscle strength, and development.

**Recommendation 4.** For children with AMC, we suggest strengthening available muscle groups to increase active range of motion, strength, mobility, stability, and improve overall health.

*Additional information*

Growth and joint contractures: Joint ROM should be monitored throughout growth, especially during growth spurts, in order to minimize worsening or recurrence of contractures. After the first three years of life, joint ROM may reach a plateau and interventions will focus more on maintaining gains and improving function, with continued stretching and orthotic use. Play activities are important to engage the child and facilitate treatment (e.g., for stretching or strengthening) through meaningful activities. Setting up specific goals for treatment will help determine effectiveness and duration of treatment. Please refer to additional information remarks in the previous section for contraindications to stretching.

Strengthening: in young children, strengthening can performed through play by stimulating limb movements in various positions (e.g., prone, sitting, standing), in gravity eliminated planes or against gravity. In older children/adolescents, encourage strengthening of available muscle groups that show an ability to contract (e.g., through active range of motion (AROM), resistive exercises, weight bearing), in order to maximize their strength and help with function (e.g., ability to transfer, stand, weight bear on upper extremities, complete self-care tasks etc.). It is important to note that in some cases, strengthening is not feasible as certain muscles may be absent or underdeveloped in AMC. Strengthening is also limited by the amount of available ROM. Aquatic therapy, when available, may be considered as an alternative way to encourage movement and strengthening. Cognitive involvement (e.g., difficulty understanding and following instructions) and lack of motivation may limit the ability to carry out specific strengthening exercise programs and these may need to be adjusted accordingly.

*Studies considered*

Please refer to the summary of evidence of recommendation #1. One review article provides recommendations on progression of ROM and strengthening interventions from birth to adolescence in children with AMC such as close monitoring of orthoses to progress ROM and therapeutic interventions following surgery to enhance joint alignment and functional ability^17^. It highlighted the importance for children with AMC to continuously stretch, improve active and passive ROM, and strengthen their muscles to help foster specific developmental activities that are tailored to the child’s needs^17^. One case report on a 9-year-old boy with AMC mentioned the use of lower limb strengthening and mobilization exercises in combination with a specific orthotic regimen over one year and reported improvement in hip and knee ROM and gait^19^. However, they considered that the effect of orthosis wear was stronger than the exercise program. Furthermore, strengthening interventions in older children are often mentioned in surgical papers, where strengthening is used post-operatively^20-23^. The benefits of strengthening on physical and mental health, muscle strength, function, and pain have been documented in children with physical disabilities, including musculoskeletal conditions^24-29^.

1. **Orthotics**

**Recommendation 5.** For children with AMC, we suggest using orthotics for the upper and/or lower limbs starting in the first year of life and during the life span to improve joint positioning, improve and maintain range of motion, provide joint alignment and stability for standing, walking, and other functional tasks, and maintain correction post-surgery or post- serial casting.

**Recommendation 6.** For children with AMC, exoskeletons for the upper limb may be used to increase function, but there is insufficient evidence to support or reject their use for upper limbs at the current time.

*Additional information*

Orthotics should be considered early in the presence of a joint contracture, starting in the first year of life. The schedule for orthotic wear (i.e., day / night) and type of orthotics should be decided according to the purpose of the orthosis, the treatment goal and the child’s needs, with the collaboration of a multidisciplinary team and the family.

Upper limb orthoses: these may include static or dynamic elbow, wrist cock-up, resting hand and thumb spica orthoses. Upper limb orthoses may be used to maintain or increase ROM (static or static progressive elbow, wrist, hand or thumb orthoses), or can be specifically used to facilitate certain tasks (e.g. self-care, leisure) when upper limb function is limited (e.g., orthosis to keep thumb out of palm, dynamic elbow orthosis to facilitate flexion). Orthoses may need to be modified or replaced as ROM changes over time and the child grows. Orthoses used to improve or maintain ROM should be started in the first year of life, continue throughout growth, and be used at night or otherwise specified in the context of surgery. Orthoses used to support function should be used during the day.

Lower limb orthoses: these may include foot orthoses (FO), ankle-foot orthosis (AFO), Knee-ankle-foot orthosis (KAFO), hip-knee-ankle-foot orthosis (HAKFO), ground reaction AFO, carbon fiber spring orthoses, knee extension orthosis, heel lift, or serial splinting. Of importance, if considering a carbon fiber spring orthosis, therapists should be aware that this type of orthotics is currently costly. A pre-requisite for this type of orthosis is a minimum of 5 degrees of ankle dorsiflexion and plantarflexion as well as a neutral alignment of the ankle. Lower limb orthoses are mainly used during the day to support standing, walking, and functional training by maintaining proper joint alignment. Night orthoses for the lower limbs are mainly used post-operatively to maintain and increase ROM achieved through surgery.

Post-operative use: Orthoses are very important post-operatively or following serial casting to maintain the gains achieved through surgery over time (e.g., elbow flexion orthosis post elbow release surgery, knee extension orthosis post knee flexion contracture release). It is important to balance orthosis wear with strengthening and functional activities with orthotics off. In addition, for children who use their feet to control their wheelchair or perform other activities, orthotic use should not hinder their capacity to do so. The timing and duration of orthotic wear is determined by the post-operative protocol and the duration of the healing process.

Other considerations: In order to ensure acceptance, promote proper use of orthotics (donning, doffing) and avoid adverse effects of orthosis wear (pressure points, improper positioning and strapping), education should be provided to the patients, caregivers and caretakers (e.g. educators, coaches). If too many orthoses are recommended at once, discomfort may be present and compliance with orthosis wear may decrease. When recommending orthotics, one should consider cost, insurance coverage, and access to orthotic services and availability of resources, which may vary with geographical location. Health care providers should advocate for access and coverage of orthotics, considering their importance for children with AMC.

Exoskeletons: The use of exoskeletons in the upper limbs to increase function in self-care and school participation has been reported in a few case reports in AMC and with children with neuromuscular diseases, as well as anecdotally. However, they are not often used clinically due to cost, size, weight and adaptability in different environments. Although there is insufficient data to make a formal recommendation for their use with children with AMC, exoskeletons can be considered when available. Children who have limited antigravity shoulder flexion but sufficient passive flexion could benefit from the support provided by the exoskeleton to encourage grasping and functional use of the upper limbs against gravity (e.g., for play, feeding or school tasks).

*Studies considered*

Five case reports on children with AMC demonstrated how upper extremity orthotics with other conservative treatments (e.g., stretching, ROM exercises) lead to improvements in joint stability and ROM, functional independence, and prevention of deformity recurrence^1-3,30-31^. In one case series^32^, the effectiveness of splint therapy for children with congenital clasped thumb and AMC was difficult to assess. However, individuals with congenital clasped thumb either with or without contractures of the thumb treated with splinting alone demonstrated improvement in thumb extension and abduction. In two case reports, children with AMC used a dynamic elbow orthosis, which lead to improvement in elbow ROM and hand-to-mouth patterns for self-feeding^6,33^. In a pilot study, serial splinting improved knee ROM in two children with knee flexion contractures^5^. In a case study on a child with AMC, the use of an overnight hip and knee extension orthosis and daytime KAFO as well as strengthening and mobilization exercises helped improve hip and knee ROM^19^. Moreover, a case series comparing gait patterns in children with AMC showed that despite the presence of joint contractures and muscle weakness, with the appropriate orthotic device, children can achieve ambulation if hip flexion contracture and weakness are minimized^34^. A case series of children with plantarflexor weakness, including myelomeningocele, arthrogryposis, and neuropathy, used a 3D gait analysis to provide evidence that a carbon fiber spring orthosis (CFO) enhances gait function in most participants by improving ankle plantarflexion moment, ankle positive work, and stride length when compared to the participants’ regular orthosis^35^. Additionally, a case series on children with myelomeningocele showed that the CFO provided functional improvement with more physiological ankle and knee kinematics compared to more standard AFOs^36^. Factors that can affect satisfaction and compliance with orthosis use include the weight of the device, its comfort, and the feeling of safety while wearing it^37^. Two review articles reemphasize the importance of using orthotics in children with AMC to maintain or correct joint mobility and prevent recurrent deformities^7,12^. The use of orthotics post-operatively is described in a later section. Six studies discuss the benefits of the use of upper extremity exoskeleton on function. A case study^38^ on an infant with Amyoplasia and a prospective case control study^39^ on children with muscle weakness, including a child with AMC, demonstrate the effects of using a passive exoskeleton for the upper limbs known as the Wilmington Robotic Exoskeleton (WREX). In both studies, improvements in UE functions were indicated, such as improved reaching and manual abilities, which allowed the patients to be more functionally independent. In a single-subject design^40^ and a case report^41^, a Playskin LiftTM (Playskin) exoskeletal garment that provides variable levels of antigravity assistance was used on toddlers with AMC. It allowed for significant improvement in their UE functions and independent ability to lift objects from the floor, manipulate objects using one hand, and couple visual and manual behavior. In a case report, children with AMC used the WREX and demonstrated improvements in various occupations such as greater independence during self-feeding and writing, as well as increased participation at school by being able to raise their hands^41^. A case study on children with neuromuscular diseases who completed the Jebsen hand function test before and after wearing the WREX for two weeks, found improvements in ADLs and ROM in their arms^43^. Additionally, the activities that participants felt benefited the most from using the WREX were "eating, stretching, increasing ROM, and raising one's hand."

1. **Mobility training and assistive equipment**

**Recommendation 7**. For children with AMC, we suggest early mobility training, including use of mobility aids and orthotics as needed, to maximize mobility (e.g., floor mobility, standing, transferring, walking, assisted walking or wheeled mobility) within their environment based on the child’s age and functional needs.

*Additional information*

Early mobility: Mobility, starting in the first year of life, should be encouraged by improving ROM, muscle strength, motor skills development and balance, and progressed according to the child’s individual capacities, functional and environmental needs (e.g., home, school, community). Standing should be initiated at around 1 year of age (this may vary depending on the child’s capacities), with the use of mobility aids when needed (e.g., standers, gait trainer, walker, push toy) and should be encouraged daily. Head control and independent sitting are an important milestone prior to advancing to standing and walking. If walking abilities are limited, introduce the use of a wheelchair at around 2.5-3 years of age (consider starting earlier as there may be delays in obtaining funding and equipment). Wheeled mobility such as adapted toy cars, may be used earlier to encourage the child to explore their environment, while working on ambulation when possible. It is important to continue working on maximizing potential for standing and/or walking even if wheeled mobility is used, considering individual and family preferences or potential. Powered mobility should be considered when self-propulsion in a manual wheelchair is challenging due to limited upper limb function and endurance. When protective reactions are limited due to upper limb impairments, the use of a soft helmet may be considered while the child increases their walking abilities and balance.

Mobility needs with growth: For children with limited walking endurance or as ambulation demands increase with age, consider the use of wheeled mobility (e.g. wheelchair) in certain environments (e.g., school) for the purpose of energy conservation and to maximize performance and function. Promoting the capacity for independent transfers at a young age (e.g., to wheelchair, bed, toilet, bath, car) is important in preparation for integration in school. In adolescents, it is important to maintain a healthy lifestyle, including exercising, good nutrition, and limiting unhealthy weight gain, in order to sustain the improved ROM and mobility skills from childhood, limit joint deterioration over time and decrease the amount of energy used in various activities. Obtaining a disabled parking permit may facilitate access to community services when mobility is limited.

General considerations: Factors to consider when recommending a mobility aid are cost, insurance coverage, acceptance, environmental accessibility, ability to transport the device and timing. The factors vary depending on geographical location. Access to mobility aids may be limited in certain regions with fewer services. Health care providers should advocate for access and coverage of such equipment as it is crucial for children with AMC. Impairments in the upper limbs must be taken into consideration when determining the type of mobility aid that is recommended (e.g. adapting the walker, using lightweight wheelchairs, considering different accesses for power wheelchairs). Wheelchair positioning should be adapted to accommodate for lower limb contractures as well (e.g., opening seat angle when hip flexion is decreased). An improperly fitted wheelchair may hinder participation. Cognitive impairment may affect progression of mobility skills and learning and type of mobility aids recommended.

*Studies considered*

Two case studies on infants with Amyoplasia^1,2^ encouraged motor skill development in the first year of life with approaches listed in recommendation #1. One case study reported that motor skill acquisition was limited by the remaining ROM limitations of the infant^1^. One mixed method study looked at the use of an adapted toy car as an introduction to early powered mobility with a 1-year-old child with AMC and showed an improvement in his ability to explore the environment and interact socially^44^. No other studies evaluated rehabilitation interventions targeting mobility in AMC. One review article discussed rehabilitation interventions to promote independent ambulation and/or mobility skills throughout the lifespan^17^. Three studies on the gait pattern in children with AMC and other pediatric orthopedic pathologies reinforced the importance of ROM and strengthening interventions, mainly of the hip, in order to improve gait and reduce compensatory trunk movements^34,45-46^.

1. **Participation in areas of life (self-care, school, work, leisure, domestic and social)**

**Recommendation 8.** For children with AMC, we suggest maximizing autonomy in self-care activities (feeding, dressing, grooming, toileting, bathing) and other meaningful activities in diverse environments (home, school, work, community) by using a team approach and goal oriented activity-based training tailored to the child’s age and needs, including practice of different strategies, trial of assistive equipment and learning from peers.

**Recommendation 9.** For children with AMC, we suggest maximizing participation in meaningful activities (school, domestic, leisure and social activities, and work) in diverse environments (home, school, work, community) by supporting accessibility and integration through environmental modifications and advocacy.

**Recommendation 10.** For children with AMC, we suggest providing opportunities for participation in meaningful activities (school, domestic, leisure and social activities, and work) in diverse environments (home, school, work, community) by guiding families in accessing appropriate external resources.

*Additional information*

Meaningful activities: Participation can be defined as the involvement in areas of life (self-care, school, leisure, domestic and social, and work). Introducing self-care tasks starts in the toddler stage, with self-feeding. At school age, there is a greater need for autonomy in tasks such as dressing, toileting, and school related activities (e.g. writing, cutting, etc.). In adolescents, toileting and personal hygiene become very important as they impact social participation and self-esteem. Other domains such as household tasks, education, transportation, leisure activities and social interactions, and work become more predominant.

Functional goals: The choice of activity depends on the child’s age and/or developmental stage, needs and interests. Functional goals should be established as a team (OT, PT, child, and caregiver) in order to ensure goals are realistic and in line with the child’s priorities. As the child ages, they should be encouraged to express their goals and needs. As functional goals of the child evolve over time, rehabilitation services should be continued or resumed as needed to address new goals, regardless of the child’s age. Youth and families should be supported in the transition towards adulthood and support the attainment of life goals (e.g., school, work, independent living). When appropriate, this preparation should include a discussion on sexuality and intimacy, living with a partner and starting a family.

Activity-based training: This refers to the process of learning a task or an activity through exploration and practice. Strategies used depend on the child’s capacities. It involves trial and error and problem solving. This can be encouraged through facilitated practice in rehabilitation sessions, or at home with the help of caregivers. Choosing a time when the family and the child are less rushed may be a good opportunity for practice of activities. Compensatory strategies, such as using external surfaces or other body parts to move the upper limb passively, can be used (e.g., for self-feeding, grooming, etc.) by children and facilitated by the OT/PT. Environmental modifications and adaptations may be required; in this case, an evaluation of the environment by an OT and/or PT is recommended.

Assistive devices: It is important that the clinician provide information about equipment and education on their benefits and use in the targeted activity. Assistive devices can be introduced to help the child learn a skill and explore, and may not be required after a certain time. Assistive devices may include everyday objects (e.g., using a hook or wall to lift pants up) or special devices (e.g., sock aid, dressing tree, universal cuff). The use of walking aids or a wheelchair may also be considered for specific activities, even if not used on a regular basis (e.g., using a wheelchair to go shopping or to play a sport). Some elements to consider when recommending equipment is acceptance by the child and the family, cost, and ease of transport and use. Although there is currently little evidence to support the use of exoskeletons to improve participation, these may be considered if available. Barriers to using them include high cost, size, weight, and adaptability in different environments.

Community support and advocacy: Ensuring the child has the opportunity to practice and integrate new skills in their different environments (e.g., home, school, community) and collaborating with involved individuals (e.g., caregivers, teachers, coach, and community organizations) is important. Learning from peers through social media, support groups, or by meeting adults with similar conditions, can be helpful to share strategies, resources and ideas (self-care, independent living, leisure activities, intimacy, starting a family, etc.). Support should be provided to facilitate access to leisure activities, with or without adaptations as needed (e.g., art, music, adaptive sports or recreation, and adapted camps). Collaboration with the school is also essential to provide education and ensure the child is provided with the appropriate time, equipment, environment adaptation and assistance needed. As the child transitions to adolescence and young adulthood towards an active life, activities must be considered in various settings (e.g. home, school, workplace, social activities) and strategies adjusted according to the youth’s preferences and abilities. Some may not be able to achieve independence in certain activities and will require assistance from another person. In such cases, it is important to teach the youth to advocate for themselves and learn how to ask for help when needed.

External resources: Various government and community resources may be available to help individuals with AMC access services and financial support for participation in different environments. The availability of resources varies depending on the geographical region and/or country. In some cases, in order to meet the youth needs, costs may need to be absorbed by the family depending on specific type of preferential therapies such as aquatic therapy, private care, hippotherapy, etc. Subsidies depend on the severity of the diagnosis and the impact on activities of daily living (e.g., Federal tax credits). It is important for clinicians to be aware of such resources and to adequately orient families. Examples of government and community resources include government financial aids for children and young adults with disabilities, access to providers of assistive devices/orthotics, school resources (e.g., specialized class, accessible schools, educational assistants, special equipment, offices for students with disabilities etc.), life skills and work programs, resources for independent living, professional counselling, community organization, adapted sports and activities, and support groups. A social worker may be able to help the family navigate governmental and community service programs.

*Studies considered*

As previously mentioned in recommendation #6, in a single-subject design^40^ and case report^41^, participation in children with AMC increased in terms of play and exploration of objects with the use of a Playskin exoskeletal garment. In a case study on an infant with Amyoplasia using specific conservative treatments listed in recommendation #1, participation in play increased due to more reaching, grasping, and being able to bring hands together at midline to play with toys and transfer them from hand to hand^1^. A mixed methods study on a child with AMC used a modified toy car as an early assisted mobility device, which allowed for an increased curiosity and desire for exploration, play, communication, motivation, and independent mobility^44^. Similarly, in a case report on a child with Amyoplasia, assistive technology was used to promote participation in meaningful occupations such as academic performance, communication, and mobility^47^. In another case report using assistive technology, children with AMC used a personalized simple feeding device and toileting ramp, which allowed for increased independence in self-care activities by improving their ability to self-feed and transfer to the toilet^48^. A review article describes various approaches that can be used to enhance participation across each age group for individuals with AMC^17^. For example, infants using adapted seating systems for upright posture to enable developmental, visual, and social skills; toddlers using gait training devices and orthotics to promote walking and exploratory skills; school-aged children using wheelchairs and canes for ambulation to increase school and social participation; and teenagers using strength and cardiovascular exercises to carry out ADLs with more ease. In a chapter by Staheli and colleagues (1999), interventions for children with AMC to promote participation in self-care activities are emphasized^49^. Interventions such as adaptive aids that increase the diameter of a spoon or fork for easier grasping, dressing frames that make it easier to slip into clothes, and toilet grab bars that help with toilet transfers all foster independence in self-feeding, dressing, and toileting, respectively. Indirect evidence from a thesis on youth with physical disabilities, including a child with AMC, demonstrates that extrinsic motivation from coaches, family members, and friends encourages children to initiate participation in recreational sports^50^. The benefits of engaging in recreational sports and the social interactions that develop can help to reduce anxiety, shyness, and peer victimization.

1. **Pain management**

**Recommendation 11.** For children with AMC, we suggest evaluating the presence (location, intensity) and type of pain, and its impact on function, in order to tailor the pain management plan.

**Recommendation 12.** For children with AMC we suggest providing treatment approaches (e.g. soft tissue management, thermal modalities, positioning, energy conservation), orthoses or mobility aids (e.g., walking aids, wheelchair), and/or a home exercise program, based on the child’s needs and tolerance, in order to reduce and/or manage pain.

**Recommendation 13.** For children with AMC, we suggest offering structured education on the concept of pain and pain management, encouraging self-management strategies, participation in support groups, and facilitating peer-to-peer support, in order to recognize, manage and/or reduce pain.

*Additional information*

Pain evaluation: Pain is defined as “An unpleasant sensory and emotional experience associated with, or resembling that associated with, actual or potential tissue damage” (IASP, 2020). Proper evaluation of the pain status is required to tailor treatment interventions to the child’s need. Thus, clinicians should have a good understanding of the type of pain and its impact on function. Clinicians should consider using validated pain scales adapted to the age of the child and pain related disability outcome measures to assess the impact of pain on function. The presence of pain may be harder to recognize in younger children. Pain may be present during stretching, casting, and after surgical interventions. Therefore, a multidisciplinary approach (rehabilitation, surgeon, nurse, caregiver) is needed to identify the best strategies to manage the pain.

Types of pain and treatment options: Pain may be related to injury of the musculoskeletal system (nociceptive pain), or may be related to nervous system involvement (neuropathic pain), and dictates pain management strategies. Thermal modalities (use of heat or cold) may help with acute pain. Evaluation of the child’s ergonomics performing the task that is causing pain may inform recommendations to improve positioning, support specific joints using orthotics or to use assistive devices, in order to decrease strain on the affected limb. Poorly fitting orthotics may contribute to pain as well, and orthoses should be adjusted or remolded accordingly. Stretching is recommended on a daily basis throughout growth, but a more specific stretching program may be added if muscle tightness appears to be contributing to pain. A strengthening program focusing on specific muscles and/or a general conditioning program can be indicated if weakness is a contributing factor to pain. Kinesiology tape may be used for overuse type of injuries (e.g., patellofemoral tracking, plantar fasciitis). When fatigue is a contributing factor, energy conservation techniques and the use of mobility aids should be considered for more energy demanding activities. Children who complain of neuropathic pain should be referred to a physician for further assessment and treatment. Once a diagnosis is established, central nervous system originating pain may be more difficult to address with the above-mentioned modalities. Peripheral nervous system originating pain, when due to mechanical compressive factors, may be improved by changes in positioning, orthoses, and stretching modalities.

Timing for referral: Referral to healthcare specialists may be indicated when the pain is chronic, has an impact on function, or fails to respond to the different modalities, and may require use of medication. A referral may also be indicated if there is a change in clinical status or function, worsening of the pain, when pharmacological treatment is indicated or for a second opinion prior to considering surgery to address pain. Specialists may include physiatrists (or physical medicine and rehabilitation specialists), orthopedists, neurologists, anesthesiologists, developmental pediatricians, or physiotherapists with expertise in pain management. Psychosocial support and coping strategies can have an important role in pain management as well.

Education, self-management and peer support: Clinicians should provide education to children with AMC and families about the different sources (e.g., surgical/iatrogenic, overuse, biomechanical misalignment) and types of pain (e.g., Nociceptive, neuropathic). Within the same individual and among different individuals with AMC, pain may vary in location, type and intensity and have an impact on function in daily activities. Self-management refers to the ability to take care of oneself. In the context of pain, we refer to self-management as the strategies that the individual knows and is able to use in order to manage their pain and be able to function throughout the day (e.g., energy conservation techniques, use of assistive devices or mobility aids when needed, use of modalities, positioning, etc.). It involves knowing what strategies work for the individual. In different contexts (e.g., home, school, rehabilitation) children should be empowered to communicate the need to use these self-management strategies. Sharing and learning from peer’s experiences (i.e., among children, youth and caregivers) is strongly encouraged in order to try new strategies and find what works for the child.

Alternative options: Alternative treatment options (e.g., hydrotherapy, acupuncture, diet, and vitamin D supplements) may be considered and should be discussed with a healthcare professional. There is insufficient evidence to support specific diets (e.g., plant based or gluten free) for pain management in children with AMC, though anecdotally some people claim it may help. Families are encouraged to connect with a physician or dietician about specific diets.

*Studies considered*

There is very limited evidence to support specific interventions for pain in children with AMC. A case study on an infant with Amyoplasia used conservative treatments (e.g., OT/PT, stretching and strengthening exercises, orthotics), which allowed for an improvement in pain scores during passive joint ROM from 10/10 to 1/10 on the The Face, Legs, Activity, Cry, and Consolability (FLACC) Pain Scale^1^. Indirect evidence on pain management can be found in a systematic review on children with disabilities, including those with AMC, which identified and classified interventions using the traffic light system (green "do it", orange "probably do it" red " don't do it")^51^. For children with physical disabilities, the following were some of the recommended interventions that indirectly relate to pain and could be applied to AMC: home programs, kinesiotape, pain management, parent education, positioning, casting, orthotics, assistive technology, hippotherapy, and massages. No specific details were provided on the different interventions.

1. **Psychosocial wellbeing**

**Recommendation 14.** For children with AMC, to improve psychosocial wellbeing, we suggest using coping strategies, peer-to-peer support and guidance on available resources, based on individual characteristics and contextual circumstances.

*Additional information*

Assessment: Psychosocial wellbeing is a quality of life construct that includes concepts of social and emotional wellbeing, feeling of happiness and satisfaction with life, mental health, self-esteem and interpersonal factors. The involvement of a social worker may be needed to address psychosocial needs of the child and family. A thorough assessment of individual characteristics and contextual circumstances is required in order to tailor intervention strategies. This includes the family composition and dynamics, the setting or geographical area (i.e., rural vs urban), the lifestyle of the child and the family, the financial situation, and the presence of social support and relationships for the family and the child. An assessment begins with active listening of the families (stories, concerns, stresses, needs), and acknowledgement of their situation, their challenges and the positive aspects of their life. Patient reported outcome measures to evaluate anxiety, depression and social relationships may help clinicians identify areas that require further attention and refer to the appropriate professionals (e.g., social worker, psychologist) and community-based resources.

Family dynamics and social support: Within the family, it is important to consider the dynamics between caregivers, caregiver-child and siblings. Factors such as understanding the child’s condition, the expectations, navigating medical appointments, and making decisions regarding the treatment plan all have an impact on family dynamics. Clinicians should keep in mind the role of siblings and close family (e.g., grandparents) and should include them when appropriate, such as introducing the team, showing them the hospital and what happens during appointments, and showing them what to expect when their caregiver is at the hospital with their sibling. Including family or friends through virtual communications (e.g., phone or video) can be a way to help the child tolerate certain treatment interventions (e.g., casting) through this extra support or distraction.

Interventions: *Coping strategies* consist of many different approaches. There are strategies that the individuals can learn to perform themselves and do not require access to specific resources. Examples include deep breathing, visualization, meditation, mindfulness. Other coping strategies involve participation in activities as a way to improve mental wellbeing, providing the opportunity for children to socialize with others (with or without disabilities) and for caregivers to meet other families. These activities can be recreational (e.g., art, music, adaptive sports or recreation, and adapted camps) or used in a therapeutic context (e.g., hippotherapy, play therapy, music therapy). Factors limiting access to activities include geographical location, financial or environmental barriers. In addition to these strategies, self-advocacy should always be encouraged in order to empower the child to navigate life situations as they grow.
Referral to a psychologist for one-on-one counselling may be needed to better cope with particular situations (e.g., issues in school, social relationships, coping with need for surgery and treatments, etc.). *Support groups* for individuals with AMC are valuable resources. They provide opportunity for peer-to-peer support and coaching. Groups on social media are a means of sharing experiences within groups of children and caregivers. *External resources* must be considered to help families with financial, logistics or social/environmental barriers. Social workers can often help families navigate such resources, including government subsidies, tax benefits, help with travel and lodging logistics (i.e., when travelling for medical appointments) and organizations that enable access to funding or other aids (Appendix 2). Availability of resources varies depending on the geographical region and/or country.

*Studies considered*

There is little evidence to support the use of specific interventions to improve psychosocial wellbeing in children with AMC. Three studies evaluated the psychosocial wellbeing of children with AMC. A cross sectional study on children with AMC using lower limb orthoses (KAFO and AFO) demonstrated significantly lower scores compared to the healthy controls for subscales pertaining to psychosocial wellbeing such as parent impact/emotional, self-esteem, and behavior on the CHQ-PF50^37^. In a mixed methods study, a child with AMC used a toy car for early assisted mobility, which allowed for improvements in psychosocial wellbeing through qualitative data previously mentioned in recommendation #8 and quantitative data such as parent ratings of encouragement required, child motivation, and enjoyment^44^. In addition, a qualitative report on a child with AMC, participation in hydrotherapy allowed the child to increase his confidence and self-esteem^52^.

Moreover, indirect evidence from a systematic review on children with neurodevelopmental disabilities participating in naturally occurring leisure activities or therapeutic programs of leisure activities (e.g., swimming, horseback riding, aerobic exercises, organized sports) demonstrated improvements in sense of self (self-competence, self-esteem, self-perception), emotional wellbeing, and social wellbeing^53^. Additionally, a cross-sectional study on children with cerebral palsy found that leisure participation in physical activities positively contributes to psychosocial wellbeing^54^. As previously mentioned in the recommendations for participation, a thesis on children with physical disabilities demonstrated how participation in recreational sports with the social interactions that are promoted are associated with positively influencing psychosocial wellbeing^50^. Similarly, a book reiterates the positive effects that physical activity and other out-of-school activities along with its opportunities for social interactions can have on children with physical disabilities such as improved mental health and well-being^55^.

1. **Perioperative rehabilitation**

**Recommendation 15.** ***Pre-operative rehabilitation*.** For children with AMC undergoing upper or lower limb surgery, we suggest pre-operative rehabilitation, including education, equipment provision, home environment modification, combined with other interventions as needed (in person rehabilitation treatment, home exercise program, psychosocial support), to prepare the family and child for surgery and optimize the child’s joint ROM and strength.

*Additional information*

Overall, pre-operative rehabilitation several weeks before surgery is advisable to prepare the child and their family by providing education and information (e.g., use of supporting materials) about the post-operative period, including pain management, support for the family, equipment needed post-operatively, and recommendations for the home environment as needed. Depending on the surgical procedure, in person rehabilitation treatment, consisting of serial casting, stretching and/or strengthening, may be required a few weeks prior to surgery in order to maximize ROM and strength of the targeted body area/joint. In cases of muscle transfers, it is important to strengthen the muscle being transferred before surgery. In some centers, rehabilitation therapists (such as physical therapists) may be involved in casting before surgery. Pre-operative rehabilitation approaches and duration should be tailored to the child and family’s needs and goals and should be discussed together with the clinical team and the family, to determine if a full episode of pre-operative rehabilitation is needed. Factors to consider include costs of services (personal or insurance coverage) and burden on family (e.g., time off work, travel time, compliance to treatment for an extended period). A home exercise program or the use of telehealth may be sufficient in some cases, or when in person treatment is not available or feasible. Collaboration between the child, family, surgeon and therapists (in hospital and therapists in local rehabilitation centers, such as occupational and physical therapists) is essential to ensure a good knowledge and understanding of the upcoming surgery.

*Studies considered*

Three studies on children with AMC reported on the use of casting (feet, knees) prior to surgery^20,23,56^. The duration of casting was 3 weeks for the knees and varied from 4-20 weeks for the feet. There were no additional studies on AMC that described pre-operative rehabilitation interventions. There may be more publications on serial casting in AMC, but these were excluded from our scoping reviews as they were surgery focused and did not have a rehabilitation component.

Although there is a lack of evidence in AMC, the benefits of the exercise component of pre-operative rehabilitation enhancing recovery and surgical outcomes are well documented in the literature across different medical fields such as oncology, cardiology, and orthopedics^57-62^. Two randomized controlled trials^63-64^ and a cohort study^65^ demonstrate that among individuals undergoing orthopedic surgery, pre-operative exercises helped reduce pain, and improve strength, range of motion, and functional capacity before surgery leading to reduced hospital stay, quicker recovery, and improved physical function and postoperative outcomes.

Pre-operative education and psychological support are also shown to be important to motivate the patient and to reduce stress and anxiety that often comes with a surgery^66-68^. According to an editorial, preoperative rehabilitation is a multimodal program that empowers the patient by efficiently educating them and allowing them to actively participate in their own care preoperatively^69^. Therefore, interventions should be tailored to a patient’s individual needs.

**Recommendation 16. *Post-operative rehabilitation.*** For children with AMC undergoing upper or lower extremity surgery, we suggest implementing rehabilitation interventions targeting muscle and joint function (ROM, stretching, orthotics, and strengthening), and activities (activity training, standing, transferring, walking, recreational activities) and offering psychosocial support when needed, to maximize functional outcomes.

*Additional information*

After a surgery, it is critical to provide rehabilitation treatments and orthoses in order to maximize functional outcomes, protect tissue repair and healing, maintain gains over time, and manage pain. Without post-operative rehabilitation, poorer outcomes are anticipated. We refer to the post-operative rehabilitation as the interventions starting after the period of immobilization following the surgery.

Orthotics: Orthoses must be ready to be used immediately after surgery or cast removal. They may be molded pre-operatively if no shape or alignment changes are anticipated in the operated body area, or should otherwise be molded immediately after surgery prior to application of cast when possible.

Goals and targeted interventions: Rehabilitation will be guided by the post-operative indications and contraindications; therefore, communication between the surgeon, the hospital occupational and/or physical therapists and/or the treating occupational and/or physical therapists in the community is important. For upper limb surgeries, when functional improvement is expected (e.g., elbow release to facilitate feeding), functional activities should be incorporated to work on the specific goals and help the child learn new skills. When providing treatment following a lower limb surgery, consider changes in body segments and joints alignment in re-learning of motor skills. Rehabilitation may be provided as in-patient for a few days or weeks if needed and if resources permit, and should be continued as outpatient. Goal setting is important to evaluate the child’s progress and this will help determine duration of treatment, which should be continued until the child reaches their goals. Type of surgery (e.g., soft tissue vs. osteotomies), child’s needs and their environment determine onset, duration and type of rehabilitation and orthoses. Aquatic therapy, when available, may be used to facilitate and encourage movement for children who may otherwise have difficulty performing activities on land. Factors such as costs of treatment, access to services, and burden of care on families may all be limiting factors to post-operative rehabilitation. Telehealth and tele-rehabilitation may be useful to address gaps, monitor progress of goals and continue providing follow-up as needed.

*Studies considered*

Several papers on surgical interventions on the lower limb in children with AMC mention the need for post-operative rehabilitation, including ROM, stretching, and orthotic use^20-22,56,70-75^. However, the rehabilitation interventions were not detailed. More information was provided on orthosis wear regimen, and this varied according to the surgery performed. Outcomes reported pertained to the surgery and there was no comparison between groups according to the post-operative rehabilitation provided. A case report on an adult with AMC who underwent lower extremity surgery described a comprehensive post-operative rehabilitation protocol, which resulted in significant improvement in walking abilities^76^.

The studies included here discuss postoperative rehabilitation for the upper limb. In a retrospective case series^77^, and a case study^78^, children with AMC underwent a posterior elbow capsulotomy with triceps lengthening followed by postoperative interventions (e.g., immobilization, hand-to-mouth activities with passive flexion, splinting). There was improvement in elbow arc of motion, the ability to reach the mouth using passive assistance, and the ability to self-feed independently for some patients. In a retrospective chart review, children with AMC treated for elbow extension contractures were divided into two groups: posterior elbow release (release group) or simultaneous posterior elbow release and humeral rotational osteotomy (simultaneous group)^79^. The release group was immobilized with an orthosis for 2-3 and the simultaneous group in a long-arm cast for 2-6 weeks. After immobilization, both groups started a ROM program and were given a flexion and extension orthoses for a minimum of 2 months if needed to maintain gains in ROM. Children in the release group experienced a significant increase in total arc of motion and maintained better elbow extension than those in the simultaneous group. In a retrospective case series, children with AMC underwent distal humerus external rotation osteotomy. Gentle elbow ROM was initiated at 2 weeks with a sling worn for the following 6 weeks, except for bathing and exercises. Weight bearing was initiated at 2 months after surgery. The postoperative plan was modified based on concomitant surgeries. At one year post-operatively, there was improvement in shoulder external rotation, better resting posture with palms facing each other, and improved writing ability for some of the participants^80^. In two case series (Carroll et al., 1970; Chomiak et al., 2014) of children with AMC who either had a triceps transfer or a pectoralis major transfer to restore elbow flexion followed by postoperative rehabilitation interventions, improvements in elbow flexion was variable^81-82^. In Carroll and colleagues’ study, the elbow was immobilized for 4 weeks using a splint then active flexion exercises begun at 4 weeks^81^. In Chomiak and colleagues’, transcutaneous electric stimulation was performed on the transferred muscle as well as elbow and shoulder exercises with isotonic concentric contractions for 4-12 weeks, in addition to by daily passive and active elbow exercises (at least 15 minutes daily for 3 months)^82^.

In a retrospective chart review^83^ and a case series^84^, children with AMC underwent a bipolar latissimus dorsi transfer to restore elbow flexion, followed by the use of an orthosis and physiotherapy, which resulted in an increased ability to perform ADLs and improvements in active elbow flexion against gravity. In Frizzell and colleagues’ study^83^, a posterior long-arm orthosis was used for 4-6 weeks, followed by therapy and the use of a locking hinged elbow brace. In Zargarbashi and colleagues’ study^84^, the elbow was casted for 6 weeks, followed by the use of a posterior splint at night for 6 weeks and physiotherapy including active mobilization and passive elbow extension. In a case study, a child with AMC underwent 1 year of preoperative occupational hand therapy, followed by a gracilis muscle transfer to restore elbow flexion, a postoperative long-arm posterior splint and hand therapy at 8 weeks with the use of muscle stimulation^85^. The child achieved a muscle grade of M4 from 0 and increased his active arc of motion of the elbow from 25 to 140 degrees. He also showed improvements in ADL performance. In a case series, children with AMC who received a median to musculocutaneous nerve transfer followed by active and passive exercises and physiotherapy initiated 1 week after surgery, resulted in two extremities reaching elbow flexion motor-grade M4, two extremities reaching M3, and one extremity reaching M1^86^. In another case series, children with AMC underwent surgical correction of thumb deformities and use of postoperative orthoses, which resulted in an increased and maintained thumb webspace, and a stable metacarpophalangeal joint was achieved in 3 out of 4 patients^87^.

**References:**

1. Azbell K, Dannemiller L. A case report of an infant with arthrogryposis. Pediatric Physical Therapy. 2015; 27(3):293-301.
2. Sala DA, Rosenthal DL, Grant AD. Early Treatment of an Infant with Sever Arthrogryposis. Physical & Occupational Therapy in Pediatrics. 1996 Jan 1; 16(3):73-90.
3. Palmer PM, Macewen GD, Bowen JR, Mathews PA. Passive motion therapy for infants with arthrogryposis. Clinical Orthopaedics and Related Research®. 1985 Apr 1; 194:54-9.
4. Binkiewicz-Glinska A, Sobierajska-Rek A, Bakula S, Wierzba J, Drewek K, Kowalski IM, Zaborowska-Sapeta K. Arthrogryposis in infancy, multidisciplinary approach: case report. BMC pediatrics. 2013 Dec; 13(1):1-6.
5. Gür G, Erel S, Yakut Y, Aksoy C, Uygur F. One-year follow-up study of serial orthotic treatment in two cases with arthrogrypotic syndromes who have bilateral knee flexion contractures. Prosthetics and Orthotics International. 2016 Jun; 40(3):388-93.
6. Kamil NI, Correia AM. A dynamic elbow flexion splint for an infant with arthrogryposis. The American Journal of Occupational Therapy. 1990 May 1; 44(5):460-1.
7. Bernstein RM. Arthrogryposis and amyoplasia. JAAOS-Journal of the American Academy of Orthopaedic Surgeons. 2002 Nov 1;1 0(6):417-24.
8. Binkiewicz-Glińska A, Wierzba J, Szurowska E, Ruckeman-Dziurdzińska K, Bakuła S, Sokołów M, Reńska A. Arthrogryposis multiplex congenital-multidisciplinary care-including own experience. Dev Period Med. 2016 Jan 1; 20(3):191-6.
9. Hamdy R, Dahan-Oliel N. Arthrogryposis. Pediatric lower limb deformities: Principles and techniques of management. 2016:297-311.
10. Hamdy RC, van Bosse H, Altiok H, Abu‐Dalu K, Kotlarsky P, Fafara A, Eidelman M. Treatment and outcomes of arthrogryposis in the lower extremity. In American Journal of Medical Genetics Part C: Seminars in Medical Genetics 2019 Sep (Vol. 181, No. 3, pp. 372-384). Hoboken, USA: John Wiley & Sons, Inc.
11. Kowalczyk B, Feluś J. Treatment of foot deformities in arthrogryposis multiplex congenita. JBJS reviews. 2015 Jun 30; 3(6):e4.
12. Kowalczyk B, Feluś J. Arthrogryposis: an update on clinical aspects, etiology, and treatment strategies. Archives of Medical Science. 2016 Feb 1; 12(1):10-24.
13. Sells JM, Jaffe KM, Hall JG. Amyoplasia, the most common type of arthrogryposis: the potential for good outcome. Pediatrics. 1996 Feb 1; 97(2):225-31.
14. Song K. Lower extremity deformity management in amyoplasia: When and how. Journal of Pediatric Orthopaedics. 2017 Sep 1; 37:S42-7.
15. van Bosse HJ. Syndromic feet: arthrogryposis and myelomeningocele. Foot and ankle clinics. 2015 Dec 1;20(4):619-44.
16. van Bosse HJ, Pontén E, Wada A, Agranovich OE, Kowalczyk B, Lebel E, Senaran H, Derevianko DV, Vavilov MA, Petrova EV, Barsukov DB. Treatment of the lower extremity contracture/deformities. Journal of Pediatric Orthopaedics. 2017 Jul 1; 37:S16-23.
17. Wagner LV, Cherry JS, Sawatzky BJ, Fąfara A, Elfassy C, Eriksson M, Montpetit K, Bucci T, Donohoe M. Rehabilitation across the lifespan for individuals with arthrogryposis. In American Journal of Medical Genetics Part C: Seminars in Medical Genetics 2019 Sep (Vol. 181, No. 3, pp. 385-392). Hoboken, USA: John Wiley & Sons, Inc.
18. Hadders‐Algra M, Boxum AG, Hielkema T, Hamer EG. Effect of early intervention in infants at very high risk of cerebral palsy: a systematic review. Developmental Medicine & Child Neurology. 2017 Mar; 59(3):246-58.
19. Moore P, Major R, Stallard J, Butler PB. Contracture correction device for arthrogryposis. Physiotherapy. 1990 May 10; 76(5):303-5.
20. Schwering L. Surgical correction of the true vertical talus deformity. Operative Orthopädie und Traumatologie. 2005 Jun; 17:211-31.
21. Fucs PM, Svartman C, de Assumpcao RM, Verde SR. Quadricepsplasty in arthrogryposis (amyoplasia): long-term follow-up. Journal of Pediatric Orthopaedics B. 2005 May 1;14(3):219-24.
22. Leonchuk SS, Novikov KI, Subramanyam KN, Shikhaleva NG, Pliev MK, Mundargi AV. Management of severe congenital flexion deformity of the knee using Ilizarov method. Journal of Pediatric Orthopaedics B. 2020 Jan 1; 29(1):47-52.
23. Moghadam MH, Birjandi, Nejad A, Ghoreishi SA. Assessment of outcome of Quadricepsplasty in Knee contractures of patients with arthrogryposis. Bangladesh Journal of Medical Science. 2015 Oct 1; 14(4).
24. Baydogan SN, Tarakci E, Kasapcopur O. Effect of strengthening versus balance-proprioceptive exercises on lower extremity function in patients with juvenile idiopathic arthritis: a randomized, single-blind clinical trial. American journal of physical medicine & rehabilitation. 2015 Jun 1; 94(6):417-28.
25. Elnaggar RK, Elshafey MA. Effects of combined resistive underwater exercises and interferential current therapy in patients with juvenile idiopathic arthritis: a randomized controlled trial. American journal of physical medicine & rehabilitation. 2016 Feb 1;95(2):96-102.
26. Gannotti ME, Fuchs RK, Roberts DE, Hobbs N, Cannon IM. Health benefits of seated speed, resistance, and power training for an individual with spastic quadriplegic cerebral palsy: A case report. Journal of Pediatric Rehabilitation Medicine. 2015 Jan 1; 8(3):251-7.
27. Kemp S, Roberts I, Gamble C, Wilkinson S, Davidson JE, Baildam EM, Cleary AG, McCann LJ, Beresford MW. A randomized comparative trial of generalized vs targeted physiotherapy in the management of childhood hypermobility. Rheumatology. 2010 Feb 1; 49(2):315-25.
28. Kristensen J, Franklyn-Miller A. Resistance training in musculoskeletal rehabilitation: a systematic review. British journal of sports medicine. 2012 Aug 1; 46(10):719-26.
29. Legerlotz K. The effects of resistance training on health of children and adolescents with disabilities. American Journal of Lifestyle Medicine. 2020 Jul; 14(4):382-96.
30. Bennett JB, Hansen PE, Granberry WM, Cain TE. Surgical management of arthrogryposis in the upper extremity. Journal of Pediatric Orthopaedics. 1985 May 1; 5(3):281-6.
31. Smith DW, Drennan JC. Arthrogryposis wrist deformities: results of infantile serial casting. Journal of Pediatric Orthopaedics. 2002 Jan 1; 22(1):44-7.
32. Tsuyuguchi Y, Masada K, Kawabata H, Kawai H, Ono K. Congenital clasped thumb: a review of forty-three cases. The Journal of hand surgery. 1985 Sep 1; 10(5):613-8.
33. Wee J, Shank TM, Castro MN, Ryan LE, Costa J, Rahman T. Elbow flexion assist orthosis for arthrogryposis. In2019 IEEE 16th International Conference on Rehabilitation Robotics (ICORR) 2019 Jun 24 (pp. 494-498). IEEE.
34. Eriksson M, Bartonek Å, Pontén E, Gutierrez-Farewik EM. Gait dynamics in the wide spectrum of children with arthrogryposis: a descriptive study. BMC Musculoskeletal Disorders. 2015 Dec;16(1):1-4.
35. Bartonek Å, EM. Effects of carbon fibre spring orthoses on gait in ambulatory children with motor disorders and plantarflexor weakness. Developmental Medicine & Child Neurology. 2007 Aug; 49(8):615-20.
36. Wolf SI, Alimusaj M, Rettig O, Döderlein L. Dynamic assist by carbon fiber spring AFOs for patients with myelomeningocele. Gait & posture. 2008 Jul 1; 28(1):175-7.
37. Eriksson M, Jylli L, Villard L, Kroksmark AK, Bartonek Å. Health-related quality of life and orthosis use in a Swedish population with arthrogryposis. Prosthetics and Orthotics International. 2018 Aug; 42(4):402-9.
38. Babik I, Kokkoni E, Cunha AB, Galloway JC, Rahman T, Lobo MA. Feasibility and effectiveness of a novel exoskeleton for an infant with arm movement impairments. Pediatric physical therapy: the official publication of the Section on Pediatrics of the American Physical Therapy Association. 2016; 28(3):338.
39. Haumont T, Rahman T, Sample W, King MM, Church C, Henley J, Jayakumar S. Wilmington robotic exoskeleton: a novel device to maintain arm improvement in muscular disease. Journal of Pediatric Orthopaedics. 2011 Jul 1; 31(5):e44-9.
40. Babik I, Cunha AB, Lobo MA. Play with objects in children with arthrogryposis: Effects of intervention with the Playskin Lift™ exoskeletal garment. In American Journal of Medical Genetics Part C: Seminars in Medical Genetics 2019 Sep (Vol. 181, No. 3, pp. 393-403). Hoboken, USA: John Wiley & Sons, Inc.
41. Lobo MA, Koshy J, Hall ML, Erol O, Cao H, Buckley JM, Galloway JC, Higginson J. Playskin Lift: Development and initial testing of an exoskeletal garment to assist upper extremity mobility and function. Physical therapy. 2016 Mar 1; 96(3):390-9.
42. Rahman, T., Sample, W., Jayakumar, S., King, M. M., Wee, J. Y., Seliktar, R., ... & Clark, A. (2006). Passive exoskeletons for assisting limb movement. *Journal of rehabilitation research and development*, 43(5), 583.
43. Rahman T, Sample W, Seliktar R, Scavina MT, Clark AL, Moran K, Alexander MA. Design and testing of a functional arm orthosis in patients with neuromuscular diseases. IEEE Transactions on Neural Systems and Rehabilitation Engineering. 2007 Jun 18; 15(2):244-51.
44. Pritchard-Wiart L, Bragg E, Thompson-Hodgetts S. The Young Movers Project: a case series describing modified toy car use as an early movement option for young children with mobility limitations. Physical & occupational therapy in pediatrics. 2019 Nov 2; 39(6):598-613.
45. Böhm H, Dussa CU, Multerer C, Döderlein L. Pathological trunk motion during walking in children with Amyoplasia: Is it caused by muscular weakness or joint contractures? Research in developmental disabilities. 2013 Nov 1; 34(11):4286-92.
46. Stief F, Böhm H, Ebert C, Döderlein L, Meurer A. Effect of compensatory trunk movements on knee and hip joint loading during gait in children with different orthopedic pathologies. Gait & posture. 2014 Mar 1; 39(3):859-64.
47. Dalton C, Hoyt-Hallett G. Enablement through provision of assistive technology: case reports of two children with physical disabilities. British Journal of Occupational Therapy. 2013 Feb;76(2):108-11.
48. Hall KW, Hammock M. Feeding and toileting devices for a child with arthrogryposis. The American Journal of Occupational Therapy: Official Publication of the American Occupational Therapy Association. 1979 Oct 1; 33(10):644-7.
49. Staheli LT, editor. Arthrogryposis: a text atlas. Cambridge university press; 1998 Apr 28.
50. Orr K. Youth sport participation and peer support in the context of physical disability: A qualitative perspective. University of Toronto (Canada); 2016.
51. Novak I, Morgan C, Fahey M, Finch-Edmondson M, Galea C, Hines A, Langdon K, Namara MM, Paton MC, Popat H, Shore B. State of the evidence traffic lights 2019: systematic review of interventions for preventing and treating children with cerebral palsy. Current neurology and neuroscience reports. 2020 Feb; 20:1-21.
52. Campbell C. Hydrotherapy: A way for exceptional children to swim toward success. A young boy with special needs learns confidence and independence through a special swimming program. Exceptional Parent. 2005; 35(6):26.
53. Dahan-Oliel N, Shikako-Thomas K, Majnemer A. Quality of life and leisure participation in children with neurodevelopmental disabilities: a thematic analysis of the literature. Quality of Life Research. 2012 Apr; 21:427-39.
54. Shikako-Thomas K, Dahan-Oliel N, Shevell M, Law M, Birnbaum R, Rosenbaum P, Poulin C, Majnemer A. Play and be happy? Leisure participation and quality of life in school-aged children with cerebral palsy. International journal of pediatrics. 2012 Oct; 2012.
55. Petrenchik TM, King GA. Pathways to positive development: Childhood participation in everyday places and activities. Mental health promotion, prevention, and intervention in children and youth: A guiding framework for occupational therapy. 2011:71-94.
56. Niki H, Staheli LT, Mosca VS. Management of clubfoot deformity in amyoplasia. Journal of Pediatric Orthopaedics. 1997 Nov 1; 17(6):803-7.
57. Blair SN, Morris JN. Healthy hearts—and the universal benefits of being physically active: physical activity and health. Annals of epidemiology. 2009 Apr 1;19(4):253-6.
58. Ditmyer MM, Topp R, Pifer M. Prehabilitation in preparation for orthopaedic surgery. Orthopaedic Nursing. 2002 Sep 1; 21(5):43-54.
59. Marmelo F, Rocha V, Moreira-Gonçalves D. The impact of prehabilitation on post-surgical complications in patients undergoing non-urgent cardiovascular surgical intervention: systematic review and meta-analysis. European journal of preventive cardiology. 2018 Mar 1;25(4):404-17.
60. Moran J, Guinan E, McCormick P, Larkin J, Mockler D, Hussey J, Moriarty J, Wilson F. The ability of prehabilitation to influence postoperative outcome after intra-abdominal operation: a systematic review and meta-analysis. Surgery. 2016 Nov 1; 160(5):1189-201.
61. Santa Mina D, Clarke H, Ritvo P, Leung YW, Matthew AG, Katz J, Trachtenberg J, Alibhai SM. Effect of total-body prehabilitation on postoperative outcomes: a systematic review and meta-analysis. Physiotherapy. 2014 Sep 1; 100(3):196-207.
62. Sebio Garcia R, Yanez Brage MI, Gimenez Moolhuyzen E, Granger CL, Denehy L. Functional and postoperative outcomes after preoperative exercise training in patients with lung cancer: a systematic review and meta-analysis. Interactive cardiovascular and thoracic surgery. 2016 Sep 1; 23(3):486-97.
63. Calatayud J, Casaña J, Ezzatvar Y, Jakobsen MD, Sundstrup E, Andersen LL. High-intensity preoperative training improves physical and functional recovery in the early post-operative periods after total knee arthroplasty: a randomized controlled trial. Knee Surgery, Sports Traumatology, Arthroscopy. 2017 Sep; 25:2864-72.
64. Rooks DS, Huang JI, Bierbaum BE, Bolus SA, Rubano J, Connolly CE, Alpert S, Iversen MD, Katz JN. Effect of preoperative exercise on measures of functional status in men and women undergoing total hip and knee arthroplasty. Arthritis Care & Research: Official Journal of the American College of Rheumatology. 2006 Oct 15; 55(5):700-8.
65. Desmeules F, Hall J, Woodhouse LJ. Prehabilitation improves physical function of individuals with severe disability from hip or knee osteoarthritis. Physiotherapy Canada. 2013 Apr;65(2):116-24.
66. Banugo P, Amoako D. Prehabilitation. BJA Education. 2017 Dec 1; 17(12):401-5.
67. Burgess LC, Arundel J, Wainwright TW. The effect of preoperative education on psychological, clinical and economic outcomes in elective spinal surgery: a systematic review. In Healthcare 2019 Mar 21 (Vol. 7, No. 1, p. 48). MDPI.
68. Celestin J, Edwards RR, Jamison RN. Pretreatment psychosocial variables as predictors of outcomes following lumbar surgery and spinal cord stimulation: a systematic review and literature synthesis. Pain Medicine. 2009 May 1; 10(4):639-53.
69. Wynter-Blyth V, Moorthy K. Prehabilitation: preparing patients for surgery. BMJ: British Medical Journal (Online). 2017 Aug 8; 358.
70. Asif S, Umer M, Beg R, Umar M. Operative treatment of bilateral hip dislocation in children with arthrogryposis multiplex congenita. Journal of Orthopaedic Surgery. 2004 Jun; 12(1):4-9.
71. Aydin BK, Yilmaz G, Senaran H, Durgut F. Short-term results of early (before 6 months) open reduction of dislocated hips in arthrogryposis multiplex congenita. Journal of Pediatric Orthopaedics B. 2016 Nov 1; 25(6):509-13.
72. Church C, McGowan A, Henley J, Donohoe M, Niiler T, Shrader MW, Nichols LR. The 5-year outcome of the Ponseti method in children with idiopathic clubfoot and arthrogryposis. Journal of Pediatric Orthopaedics. 2020 Aug 7;40(7):e641-6.
73. Ho CA, Karol LA. The utility of knee releases in arthrogryposis. Journal of Pediatric Orthopaedics. 2008 Apr 1; 28(3):307-13.
74. van Bosse HJ, Feldman DS, Anavian J, Sala DA. Treatment of knee flexion contractures in patients with arthrogryposis. Journal of Pediatric Orthopaedics. 2007 Dec 1; 27(8):930-7.
75. Szöke G, Staheli LT, Jaffe K, Hall JG. Medial-approach open reduction of hip dislocation in amyoplasia-type arthrogryposis. Journal of Pediatric Orthopaedics. 1996 Jan 1; 16(1):127-30.
76. Taricco LD, Aoki SS. Rehabilitation of an adult patient with arthrogryposis multiplex congenita treated with an external fixator. American journal of physical medicine & rehabilitation. 2009 May 1; 88(5):431-4.
77. Van Heest A, James MA, Lewica A, Anderson KA. Posterior elbow capsulotomy with triceps lengthening for treatment of elbow extension contracture in children with arthrogryposis. JBJS. 2008 Jul 1; 90(7):1517-23.
78. Van Heest A, Waters PM, Simmons BP. Surgical treatment of arthrogryposis of the elbow. The Journal of hand surgery. 1998 Nov 1; 23(6):1063-70.
79. Ramirez RN, Richards CJ, Kozin SH, Zlotolow DA. Combined elbow release and humeral rotational osteotomy in arthrogryposis. The Journal of Hand Surgery. 2017 Nov 1; 42(11):926-e1.
80. Wall LB, Calhoun V, Roberts S, Goldfarb CA. Distal humerus external rotation osteotomy for hand position in arthrogryposis. The Journal of hand surgery. 2017 Jun 1; 42(6):473-e1.
81. Carroll RE, Hill NA. Triceps Transfer to Restore Elbow Flexion: a study of fifteen patients with paralytic lesions and arthrogryposis. JBJS. 1970 Mar 1; 52(2):239-44.
82. Chomiak J, Dungl P, Vcelák J. Reconstruction of elbow flexion in arthrogryposis multiplex congenita type I: results of transfer of pectoralis major muscle with follow-up at skeletal maturity. Journal of Pediatric Orthopaedics. 2014 Dec 1; 34(8):799-807.
83. Frizzell K, Kozin SH, Zlotolow DA. Bipolar latissimus dorsi transfer for arthrogryposis multiplex congenita: minimum 10-month follow-up. The Journal of Hand Surgery. 2020 Nov 1; 45(11):1084-e1.
84. Zargarbashi R, Nabian MH, Werthel JD, Valenti P. Is bipolar latissimus dorsi transfer a reliable option to restore elbow flexion in children with arthrogryposis? A review of 13 tendon transfers. Journal of Shoulder and Elbow Surgery. 2017 Nov 1; 26(11):2004-9.
85. Sochol KM, Edwards III G, Stevanovic M. Restoration of elbow flexion with a free functional gracilis muscle transfer in an arthrogrypotic patient using a motor nerve to pectoralis major. HAND. 2020 Sep; 15(5):NP80-4.
86. Hagemann C, Stücker R, Breyer S, Kunkel PO. Nerve transfer from the median to musculocutaneous nerve to induce active elbow flexion in selected cases of arthrogryposis multiplex congenita. Microsurgery. 2019 Nov; 39(8):710-4.
87. Dangles CJ, Bilos ZJ. Surgical correction of thumb deformity in arthrogryposis multiplex congenita. Hand. 1981 Feb;(1):55-8.
